# The effectiveness of a combined exercise and psychological treatment programme on measures of nervous system sensitisation in adults with chronic musculoskeletal pain - a systematic review and meta-analysis.

**DOI:** 10.1186/s12891-024-07274-8

**Published:** 2024-02-14

**Authors:** Orla Deegan, Brona M Fullen, Ricardo Segurado, Catherine Doody

**Affiliations:** School of Public Health, Physiotherapy and Sports Science, Health Sciences Building, University College Dublin, Belfield, Dublin 4, Ireland

**Keywords:** Chronic pain, Exercise, Psychological intervention, Pain sensitisation, Quantitative sensory testing

## Abstract

**Background:**

Quantitative sensory testing (QST) offers information regarding underlying mechanisms contributing to chronic pain (CP) in adults with musculoskeletal disorders. This review examined the use of QST measures in adults with CP following participation in a combined exercise and psychological intervention.

**Methods:**

The review was conducted in accordance with the PRISMA guidelines. Five databases were searched from inception to November 2022. All study designs which evaluated the effects of a combined exercise and psychological treatment on measures of nervous system sensitivity in adults with chronic musculoskeletal pain were included.

**Results:**

A total of 13 studies met the selection criteria, 10 of which were included in a meta-analysis. Local pressure pain thresholds were the most frequently used measure (*n* = 12 studies). Meta-analysis revealed statistically significantly improvements in favour of the combined exercise and psychological intervention group, compared to a control group, for local pressure pain threshold measures [SMD = 0.44, 95% CI 0.08–0.81, I^2^ = 84%], pain intensity scores [SMD=-0.89, 95% CI -1.66- -0.13, I^2^ = 94%] and the Central Sensitisation Inventory [SMD=-0.69, 95% CI -1.37- -0.02, I^2^ = 87%]. There were no significant differences found between groups for remote pressure pain thresholds, temporal summation or conditioned pain modulation.

**Conclusions:**

The results suggest that a combined exercise and psychological intervention may lead to greater improvements in local pressure pain threshold, pain intensity and Central Sensitisation Inventory scores when compared to a control intervention in adults with CP, however these findings must be interpreted with caution as a large degree of heterogeneity was present in these results (I^2^: 84–94%). Further large, longitudinal studies are required using standardised QST measurement procedures and patient reported outcome measures to explore changes in nervous system sensitisation.

**Trial registration:**

This systematic review is registered with PROSPERO, ID Number CRD42022380464.

**Supplementary Information:**

The online version contains supplementary material available at 10.1186/s12891-024-07274-8.

## Background

Chronic pain (CP) is postulated to be augmented by changes in modulation of sensory inputs by the peripheral and central nervous system [1–4]. It has been suggested that evaluation of different pain mechanisms may help to individualise and tailor pain management strategies, targeting individuals specific pain mechanisms [5].

Quantitative sensory testing (QST) may be useful for assessing pathology in pain processing. QST assessments are psychophysical methods of testing the perception of touch, vibration, proprioception, pinprick/blunt pressure sensitivity or sensitivity to cold or heat stimuli using stimuli applied under standardised testing protocols, and the participants’ self-reported sensory experience is quantified [6]. QST can offer information about the potential underlying mechanisms contributing to pain in musculoskeletal disorders and can explore mechanisms responsible for the development or maintenance of local and widespread pain [7, 8]. QST can be subdivided into static and dynamic measures [9, 10]. Static QST, for example pressure pain thresholds (PPT), typically refers to the measurement of the threshold of pain that primarily reflects states of the peripheral nervous system [9]. PPTs can be measured local to the primary pain area or at a remote site. PPTs measured at remote sites while also measuring static mechanical allodynia, when decreased are thought to reflect mechanisms of central sensitisation (CS) [11]. Dynamic QST measure mechanisms of pain processing in the central nervous system [9]. For example, conditioned pain modulation (CPM) is thought to be a measure of the brain’s capacity for activating endogenous analgesia, via the descending inhibitory tracts in the central nervous system [12] and temporal summation (TS) measures the state of hyperactivity in the dorsal horn of the pain facilitation pathways.

Information regarding individuals possible underlying pain mechanisms can also be inferred using a number of patient reported outcome measures (e.g. Pain-Detect for neuropathic pain or Central Sensitisation Inventory (CSI) for CS).

The biopsychosocial treatment model which acknowledges and addresses the biological, psychological and social contributions to CP and disability is currently seen as the most efficacious approach to the management of CP [13]. Psychological treatments designed to target potential pain mechanisms contributing to CP [14] have been utilised in pain treatment programmes [15]. In addition, physical activity is considered important for the promotion of biopsychosocial health [16] and is an important health management and disease prevention strategy for adults with CP, recommended for its neuromodulatory benefits, and is suggested for a variety of CP conditions such as arthritis [17, 18], fibromyalgia [19], and dysmenorrhoea [20]. A number of QST studies investigating the effects of exercise in populations of pain free adults have demonstrated exercise has an effect on a number of measures of nervous system sensitivity; showing lower central excitability (measured using TS), increased pain thresholds (PPT) and enhanced CPM [21–23]. More variable results have been found in QST measures following exercise in clinical populations. Oosterwijck and colleagues [24] examined 22 women with chronic whiplash associated disorder and 22 healthy controls who performed a self-paced exercise test on a cycle ergometer and found PPT decreased following submaximal exercise in the individuals with whiplash and increased in healthy subjects. Furthermore, Meeus and colleagues [25] examined the change in mean PPT in response to exercise in 26 patients with chronic fatigue syndrome with CP, 21 patients with chronic low back pain and 31 healthy subjects. After submaximal aerobic exercise, mean pain thresholds decreased in patients with chronic fatigue syndrome, and increased in the chronic low back pain and healthy subjects.

This review aims to examine the use of QST as measures of pain sensitisation in adults with chronic musculoskeletal pain who have taken part in a combined exercise and psychological intervention. The review will further investigate changes in QST measures in adults with chronic musculoskeletal pain following participation in interventions combining exercise and psychological interventions. A greater understanding of the role of QST in measuring change following participation in these programmes may help to quantify the benefits of this type of pain management programme.

## Methods

### Study design and registration

The systematic review and meta-analyses were conducted in accordance with the Cochrane Handbook for Systematic Reviews [26] and the results are reported according to the guidelines included in the Preferred Reporting Items for Systematic Reviews and Meta-Analyses (PRISMA) statement [27]. The systematic review was registered on the Prospero database (CRD42022380464 ). A protocol was not published for the current review.

### Literature search and identification of studies

To identify the relevant literature, electronic searches were conducted in the following databases: Medline (OVID), Embase (OVID), SportsDiscus (EBSCO), Scopus (Elsevier) and Cinahl (EBSCO) from their inception to 14th Nov 2022. A comprehensive search strategy was designed with the assistance of an experienced medical librarian. The search strategy was adjusted to account for differences in indexing across databases and developed using medical subject headings (MeSH) where available. The search strategy (Supplementary Material; S1) included relevant keywords which encompass terms for the three domains of interest: chronic pain, nervous system sensitization and combined exercise and psychological treatment. The focus of the review was chronic musculoskeletal pain as defined by ICD 11 for ‘Chronic Primary Musculoskeletal Pain’ and ‘Chronic Secondary Musculoskeletal Pain’. As such the review did not include TMJ disorders or chronic headache specifically. References lists of identified systematic studies were also screened. Articles obtained following the systematic search were exported and saved into an online review management platform (Rayyan) where duplicates were removed. Following removal of duplicate papers, studies titles and abstracts were screened independently for inclusion and exclusion criteria by two reviewers (OD and CD). Full texts of the remaining studies were reviewed independently by the two reviewers for their eligibility. Any disagreements arising between the reviewers were resolved by discussion and consensus and the assistance of a third reviewer was not required.

### Eligibility criteria

Only articles published as full text in the English language in peer-reviewed journals were included. Studies including adults (≥18 years) with chronic musculoskeletal pain (defined as pain persisting for ≥12 weeks) were eligible. Studies were included which investigated changes in nervous system sensitisation following interventions that included a combined exercise and psychological intervention. Studies had to be of an experimental design with an intervention and comparison control group. Studies were excluded if participants in both the intervention and control groups had pain due to serious pathologies; including fractures, neoplasm, infection, or specific conditions; rheumatoid arthritis, pregnancy, postpartum pain, or fibromyalgia. Participants who had undergone a surgical intervention in the three months prior to study participation were also excluded. Studies were excluded if surgical, injection, electro-acupuncture or oral analgesic treatments were used in the intervention or control group. The main nervous system sensitisation outcomes of interest were QST measures as well as patient reported outcome measures (questionnaires which primarily focused on measurement of nervous system sensitization).

In this review, the term QST included both static and dynamic measures of nervous system sensitization (e.g. pressure pain threshold (PPT), temporal summation (TS), conditioned pain modulation (CPM) pain tolerance threshold, thermal threshold). Questionnaires investigating nervous system sensitization were also included i.e. the CSI. All study designs were included if they utilized these measures pre and post a combined exercise and psychological treatment intervention in adults with CP. Exercise was defined as ‘a series of specific movements with the aim of training or developing the body by a routine practice or as physical training to promote good physical health’ [28]. An exercise intervention encompasses a heterogeneous set of treatments prescribed by a health professional that include conducting specific activities, postures and/or movements. The psychological intervention for the experimental group included treatment with a definable psychotherapeutic content (e.g., Cognitive Behavioural Therapy, Pain Neuroscience Education, Mindfulness-Based Stress Reduction and excluded other types of general education) delivered by a health professional.

### Evaluation of methodological quality

The methodological quality of each of the randomised control trials (RCT) in the review were assessed using the PEDro scale [29] scored by two independent reviewers. The PEDro scale’s score depends on the presence or absence of the 11 criteria related to randomization, blinding, and data treatment. Studies are rated from 0 to 3 (poor quality), 4–5 (fair quality), 6–8 (good quality) and 9–11 (excellent quality) [30, 31]. The modified Newcastle Ottawa Scale (NOS) [32] was used for other study designs e.g., case-control and cross-sectional studies. The NOS assigns up to a maximum of eight points for the least risk of bias in three domains: (1) selection of study groups (four points); (2) comparability of groups (one point); and (3) ascertainment of exposure and outcomes (three points). Studies are rated from 0 to 9, with those studies rating 0–2 (poor quality), 3–5 (fair quality), 6–9 (good/high quality) [33]. Survey questions were developed based on the NOS questions covering all three domains so that authors could provide detailed information about their studies. Studies rated as ‘fair quality’ or above were included in the review.

### Data extraction

Data from each study were extracted by two reviewers (O.D. and C.D.) independently using a customized data extraction tool in the form of an MS Excel spreadsheet. The following data were extracted: (1) study characteristics: number of participants, sex, age, duration of pain symptoms, area of pain, study inclusion criteria; (2) characteristics of the interventions for both exercise and psychological intervention: type of intervention (group vs. individual), dosage, and description of content of interventions; (3) outcome measures: type of QST measure; data per group/time-point, description of QST area, type of patient reported outcome measure; data per group/time-point, and (4) summary of findings. Studies with similar outcome measures allowed a meta-analysis to be performed. QST data were extracted in the unit presented in each paper (kPA, kg/cm^2^). All scores were then converted to kg/cm^2^ for analysis using a converter application, applying the following formula: 1 kg-force/Square Centimeter (kg/cm^2^) = 98.0665 Kilopascal (kPa). Completed data collection forms including data used in the meta-analysis are available upon request from the lead researcher (O.D.).

### Data analysis

The QST measures were sub-classified as ‘static’ and ‘dynamic’, with static modalities including PPTs, and dynamic modalities including CPM and TS. PPT measures were divided into local and remote measures, with local measures defined as those acquired from sites identified as the primary area of pain in the studied population and remote measures were defined as those acquired from sites which were not local to the primary area of pain [34]. In the event that studies performed QST at multiple local and remote sites, data were pooled according to local and remote site for meta-analysis, as recommended [35].

Pain intensity was extracted where available from the included studies. If more than one measure of pain intensity was used in a study, then the one considered to be the primary outcome or the one considered most similar to outcome measures used in other included studies was chosen for inclusion in the meta-analysis (e.g., Numerical Rating Scale and Visual Analog Scale were considered similar outcome measures as they both require the rating on an 11-point severity scale).

Group means, standard deviations (SD), and sample sizes were extracted and analysed for each follow-up timepoint. If outcomes were incompletely reported or unclear, authors were contacted via e-mail. Where data were presented in graph format only the authors were contacted via e-mail with a request to provide the raw data. In cases where authors were uncontactable, a web-based tool was used to extract the data from the graphs (https://automeris.io/WebPlotDigitizer/). A number of studies reported median and interquartile range scores. Under the assumption of normality of the underlying distribution, the median was substituted for the mean and the width of the interquartile range was used as an approximation of 1.35 times the SD [36]. If the SD was not given, it was calculated from the SE or confidence intervals when these were available. If no estimate was possible, the data were not used in the meta-analysis [36]. Where data were presented as pre-intervention mean (SD) and mean (SD) change with intervention only, the pre intervention SD was utilised for the post-intervention mean based on Cochrane guidelines [36].

Meta-analyses of study outcomes were performed where possible using RevMan (The Nordic Cochrane Centre, Copenhagen, Denmark) using a random effects model. The standard mean difference (SMD) was calculated using the scores at post-intervention for each group. Statistical heterogeneity was assessed by calculating I^2^, which represents the percentage of total variation across studies that is due to heterogeneity rather than chance [37, 38]. An I^**2**^ value of 25% represents a small, 50% a moderate, and 75% a large degree of heterogeneity [37]. Pooled findings for each outcome were reported as SMD and 95% CI. The SMD is more generalisable than the mean difference (MD), and has similar statistical power as the MD [39]. Effect size was interpreted using Cohen’s criteria for pooled estimates [40]. Cohen described 0.2 as small, 0.5 as moderate, and 0.8 as large effect sizes. Statistical significance was set at a level of 0.05.

## Results

### Study selection

**Fig. 1:**
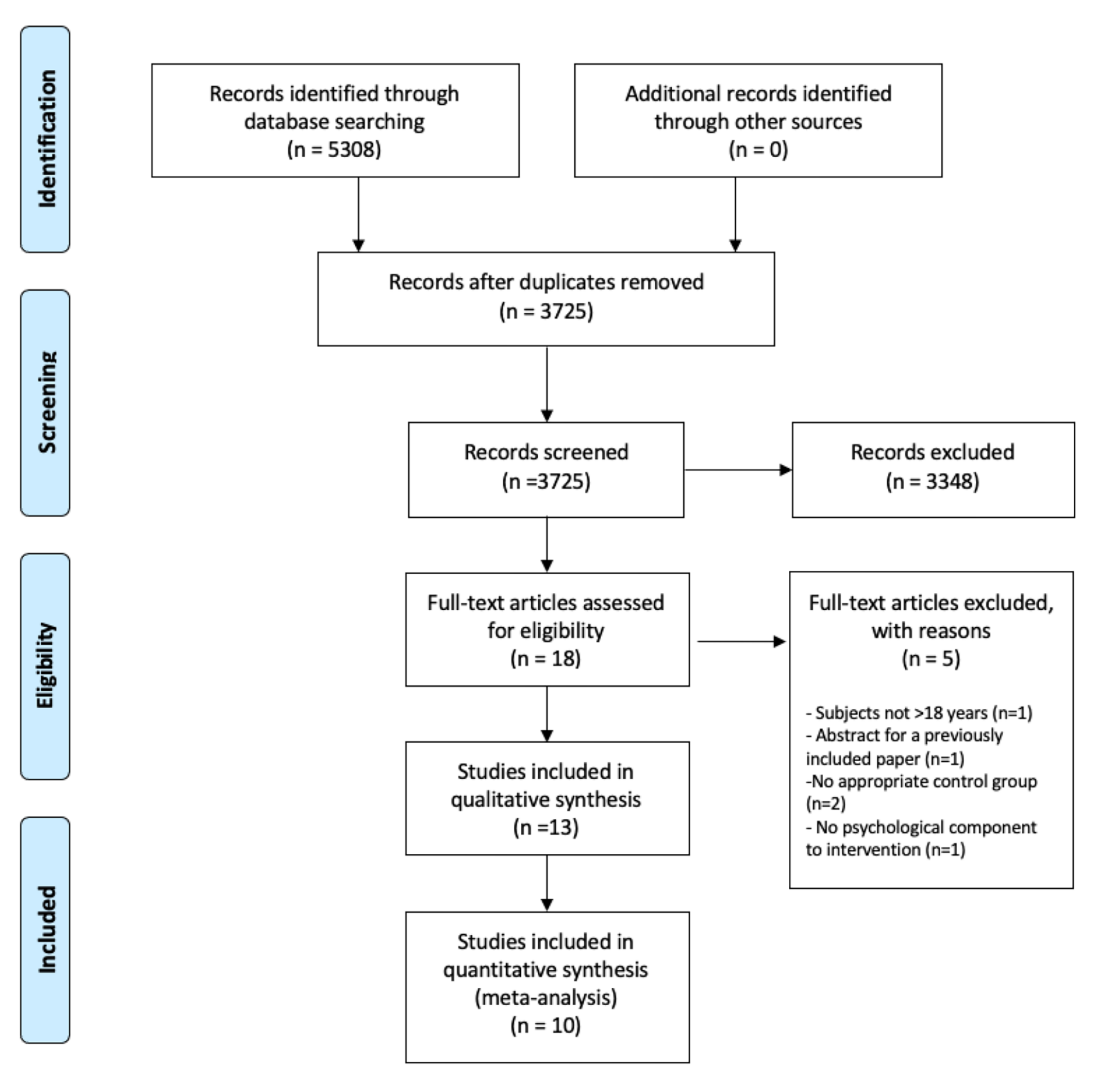
PRISMA flow diagram Legend: n, number of participants; ≥ greater than or equal to

The trial identification process is summarized in Fig. 1. The electronic database search yielded 5308 records. After removal of duplicates, 3725 were screened independently by two reviewers based on title and abstracts. A further 3707 records were excluded, leaving 18 to be screened by full text. Of these, five studies did not fit the eligibility criteria for this review. The reasons for exclusion were; the subjects included in the study were not ≥18 years old, the article reviewed was an abstract from a previously included paper, the study had no appropriate psychological intervention and the studies did not have an appropriate control group comparison. A final total of 13 records met the selection criteria [41–53]. A number of study designs were identified in the included studies; randomised controlled trials (*n* = 10) [41–50], case control study (*n* = 1) [53], non-randomised controlled trial (*n* = 1) [52], and prospective cohort study (*n* = 1) [51].

### Nervous system sensitisation measurement techniques

A description of the included studies is provided in Table 1. Twelve trials studied the effects of a combined exercise and psychological intervention on measures of local PPT [41–52] and 9 studied the effect of combined interventions on measures of remote PPT [41, 43, 45, 46, 48–52]. The study populations where PPT was performed included; chronic low back pain [41, 42, 46, 48, 52, 53], chronic neck pain [47, 49, 51], chronic spinal pain [44], knee osteoarthritis [45, 50] and chronic Achilles tendinopathy [43]. Holm et al. [45] measured PPT using a computer-controlled cuff algometer. The remaining studies measured PPT using a handheld pressure algometer device.

**Table 1:** Characteristics of included studies

Author	Population	Experimental Intervention (G1)	Control (G2)	Measure of NSS	Measurement time-points	Summary of Findings
Vaegter et al. [53]	Chronic low back pain *N* = 146 F92 M54 Age = 52.2 (13.2) years	Cognitive functional therapy: 8 supervised Rxs over 12 wks including (1) making sense of pain; (2) exposure with control; (3) lifestyle change. Encouraged to perform 20–30 min of physical activity daily based on personal preference.	Multidisciplinary intervention combining (1) medical Rx with a specialist pain consultant & (2) one or more of the following: individual consultations with a pain psychologist, or social worker with CBT Training, or participation in a group session with relaxation therapy or mindfulness.	PPT (local &remote) -kPa PPT assessed at the right erector spinae muscle (local) & left upper trapezius muscle (remote) using a handheld pressure algometer (Somedic Sales AB, Norra Melby, Sweden) with a stimulation area of 1 cm^2^, pressure rate of 30 kPa/s.	Pre Intervention Post intervention 6-mth f/up	PPT (local) Mean (IQR) Pre: G1: 183.3 (132.4–278.0) Post: G1: 230 (193.5–363.5) 6 mth f/up: G1: 257.5 (192.0-367.0) PPT (remote) Pre: G1: 189.5 (121.0-251.0) Post: G1: 208.75 (152.0-296.5) 6 mth f/up: G1: 218.0 (160.5–308.0) Sig. increase in lumbar PPTs at end of Rx. period & at 6-th f/up in CFT group. No sig. increase in remote PPTs at both time points. Moderate association between PPT lumbar & pain intensity at both time points.
Bodes-Pardo et al. [41]	Chronic low back pain *N* = 56 F 44 M 12 Age = 47.1 (10.1) years	2 group session (30–50 min) of Pain Neuroscience Education & information leaflet. Daily exercise for 3-months: multimodal exercise program consisting of motor control exercises, stretching & aerobic exercise.	Daily exercise only (as experimental group)	PPT (local & remote) -kg/cm^2^ PPT assessed 5 cm lateral to the spinous process of L3 (local) & at 2 cm from the lateral epicondyle (remote) using an analogue Fisher algometer (Force Dial model FDK 40) with a surface area of 1cm^2^. CSI	Pre intervention Post intervention 1 mth f/up	PPT (local) Mean (95% CI) Pre: G1: 2.8(2.5-3.0) Post: G1: 3.9(3.6–4.3) 1 mth f/up: G1: 4.6(4.3–4.9) Pre: G2: 3.0(2.7–3.2) Post: G2: 3.2(3.0-3.5) 1 mth f/up: G2: 3.6(3.3–3.9) PPT (remote) Pre: G1: 3.6(3.2-4.0) Post: G1: 3.6(3.2-4.0) 1 mth f/up: G1: 3.7(3.3–4.1) Pre: G2: 3.8(3.5–4.2) Post: G2: 4.0(3.6–4.3) 1 mth f-up: G2: 3.9(3.6–4.3) Sig. differences between groups for local PPT in favour of experimental group at 1 mth f/up No significant between group differences for remote PPTs at 1 mth f/up Follow up CSI scores not reported.
Sitges et al. [52]	Chronic low back pain *N* = 59 F 42 M 17 Age = 46.8 (8.4) years	Self-managed (G2) or supervised (G1): twice a wk for 4 wks: (1) pain education video < 4 min (2) 50 min exercise, strength exercises, motor control, relaxation routine, flexibility and self-massage.	nil	PPT (local & remote) -kg/cm^2^ PPT assessed unilateral erector spinae muscle, 2 cm from spine at most painful point (local) & at the forefinger (remote) using a digital algometer (FPIX 50; Wagner Instruments).	Pre intervention Post intervention	PPT (local) Mean (SD) Pre: G1: 3.49(1.17) Post: G1 3.23(1.20) Pre: G2 3.66(1.23) Post: G2: 3.39(1.18) PPT (remote) Pre: G1: 3.91(1.01) Post: G1: 3.69(1.19) Pre: G2: 4.13(1.02) Post: G2: 4.07(0.96) No sig. differences between the groups were found in local or remote PPTs post intervention.
Malfliet et al. [46]	Chronic low back pain *N* = 120 F 73 M 47 Age = 39.8 (12.4) years	Biopsychosocial Approach: Pain Neuroscience Education, Cognition-Targeted. Time‐Contingent Exercise Program, (“Perform this exercise 10 times regardless the symptoms it might induce.”) Both interventions comprised 3 educational sessions (group session, home-based online module, and individual session) & 15 one-on-one exercise sessions over 12 wks	Biomedical Approach: Traditional Back/Neck School Pain-Contingent Exercise Program (“Stop or adapt the exercise as soon as symptoms occur.”)	PPT (local, distal, remote) -kgF PPT assessed at the symptomatic sites (trapezius muscle midway between C7 & the acromion tip & 5 cm lateral of the spinous process of L3 (local) & remote sites at quadriceps muscle (distal) & the web between the thumb and index finger (remote) using a digital pressure algometer with a 1-cm^2^ tip (Wagner Instruments) CPM measured using a cold-water bath (12 °C; Versacool) for 2 minutes’ immersion of the hand contralateral to the PPT measurements. CSI	Pre intervention Post intervention	PPT (local) Mean (SD) Pre: G1: 4.56(2.40) Post: G1 6.15(2.73) Pre: G2 4.43(2.45) Post: G2: 5.18(2.81) PPT (remote) Pre: G1: 3.6(1.88) Post: G1: 4.41(2.01) Pre: G2: 3.6(1.88) Post: G2: 4.05(2.09) PPT (distal) Pre: G1: 5.33(2.57) Post: G1: 6.54(0.37) Pre: G2: 5.08(2.53) Post: G2: 5.65 (0.38) Local PPTs in experimental group showed a clinically relevant (> 15%) increase in PPTs post intervention. Nil noted in remote or distal PPTs. CPM Mean (SE) Pre: G1: 1.08(0.20) Post: G1: 1.51(0.21) Pre: G2: 1.05(0.19) Post: G2: 1.19(0.22) No sig. CPM group difference post intervention. CSI Mean (SE) Pre: G1: 40.02(1.47) Post: G1: 30.67(1.69) 6 mth f/up: G1: 25.24(1.73) 12 mth f/up: G1: 29.36(1.67) Pre: G2: 39.88(1.47) Post: G2: 35.24(1.71) 6 mth f/up: G2: 34.22(1.77) 12 mth f/up:G2: 35.14(1.70) Experimental group showed sig. lower CSI scores (medium effect sizes) than control group post intervention.
Cabak et al. [42]	Chronic back pain *N* = 68 F 49 M 19 Age = 58.8 (10.5) years	1 consultation per mth for 3 mths. (1) history taken (2) health education, individualised psychological support (3) instructions on home exercise- A set of ‘5 exercises in 5 minutes’ (4) 15 minute massage.	Waiting list	PPT (local) -kg/cm^2^ PPT assessed at selected pain trigger points on the trapezius muscle, levator scapula muscle and multifidus muscle, on both sides of the spine (local) & at quadriceps muscle & the web between the thumb & index finger (remote) using a Pain Test Algometer FPX.	Pre intervention Post intervention	PPT scores Mean (SD) Post-intervention scores only G1: Traps(L) 4.636(0.980) G1: Traps(R) 4.786(1.111) G1: Lev Scap (L) 5.533(1.209) G1: Lev Scap (R) 5.828(1.065) G1: Multifidus (L) 5.995(0.926) G1: Multifidus (R) 6.238 (0.839) G2: Traps(L) 3.363(1.281) G2: Traps(R) 3.398(1.184) G2: Lev Scap (L) 3.761(1.215) G2: Lev Scap (R) 3.894(1.137) G2: Multifidus (L) 3.813(1.263) G2: Multifidus (R) 3.798(1.293) PPT in all local muscles under analysis were sig. higher in the experimental group compared to the control group post-intervention.
Ris et al. [49]	Chronic neck pain *N* = 200 F 149 M 51 Age: 45.2 years	4 sessions (1.5 h each, once per month) focusing on understanding & acceptance of pain, goal setting, participation in social and work-related contexts based on a cognitive concept. 8 sessions of 30 min instruction in progressive individually tailored exercises (1) neck flexor and extensor function, (2) standing balance, oculomotor training & neuromuscular function of the shoulder girdle.	Pain Education (as experimental group) alone	PPT (local & remote) -kgF PPT assessed at infraspinatus and C5/6 level (local) & at anterior tibialis (remote) using an algometer (Wagner, FPX algometer, USA)	Pre intervention Post intervention	Difference of PPT change scores between groups at f-up. PPT local Mean (95% CI) Infraspinatus(L): 1.97(-2.40-6.31) Infraspinatus(R): 0.07(-0.35-0.50) Cervical (L): -0.21(-0.55-0.15) Cervical (R):-0.76(-1.76-0.24) PPT remote Tib Ant (L): -0.54(-0.94-0.15) Tib Ant (R): -0.35(-0.75-0.04) Local and remote PPT improved sig. for the experimental group compared to control.
Galan-Martin et al. [44]	Chronic spinal pain *N* = 170 F 136 M 34 Age = 51.1 (11.4) years	Pain Neuroscience Education-6 sessions (10 h) & 18 sessions of group therapeutic exercise over 6 wks	Usual physiotherapy Rx, 15 sessions (15 h) of thermotherapy & analgesic electrotherapy in the area or areas of pain, & exercises recommended by the Spanish Society of Physical Medicine & Rehabilitation	PPT (local) -kg/cm^2^ PPT assessed at 4 reference points(P1,2,3,4); midpoint between the acromion & the spinal process of the seventh cervical vertebral, bilaterally, & the midpoint between the highest part of the superior border of the iliac crest & the spinal process at the same height, also bilaterally (local) using an algometer with an application area of 1 cm^2^ (Warner Instruments FPX-100).	Pre intervention Post intervention 6 mth follow-up	PPT intragroup difference (6mth-Pre) Local PPTs Mean (95% CI) P1 G1: 1.8 (1.5 -2.2) P1 G2: 0 (-0.2-0.3) P2 G1:1.9 (1.6 -2.2) P2 G2: 0.2 (-0.1- 0.5) P3 G1: 2.4 (2- 2.8) P3 G2: 0.2(-0.1- 0.5) P4 G1: 2.5 (2- 2.9) P4 G2: 0.2 (-0.1-0.5) Sig. intragroup differences for all local PPTs between six months assessment and initial assessment and significant intergroup differences between six months assessment and initial assessment in favour of experimental group CSI scores Mean (SD) Pre: G1: 43.4(12.5) Post: G1: 25.7(10.8) 6 mth f/up: G1: 25.8(10.5) Pre: G2: 38.6(11.7) Post: G2: 37.7(12.4) 6 mth f/up: G2 37.4(13.5) At 6 mth f/up there was significant differences between groups favouring G1 for CSI scores.
Skou et al. [50]	Knee Osteoarthritis *N* = 100 F 51 M 49 Age: 65.9 (8.9) years	3-mth programme Education, 2 × 60-min -focus on disease characteristics, OA pain & how to control & monitor it during exercise, Rx & help to self-help by actively engaging the patients +& Neuromuscular Exercise training 60 min twice weekly. Optional pain medication, insoles, dietary advice	Usual care-2 leaflets with education about OA	PPT (local, remote, distal) -kPa PPT assessed at four sites at the knee, all in proximity to the patella (local) & at tibialis anterior muscle (distal) & extensor carpi radialis longus muscle (remote) using a handheld algometer with a 1 cm^2^ probe (Algometer Type II, Somedic AB, Hoerby, Sweden)	Pre intervention Post intervention	PPT local Mean (SD) Pre: G1: 521.71(241.42) Post: G1 595.79(251.23) Pre: G2: 572.80(297.16) Post: G2: 628.37(287.15) PPT remote Pre: G1: 402.99(180.86) Post: G1 415.99(198.03) Pre: G2: 401.48(234.22) Post: G2: 198.03(166.59) PPT distal Pre: G1: 575.74(295.15) Post: G1: 664.70(332.39) Pre: G2: 610.40(346.21) Post: G2: 670.69(293.95) No statistical difference in change in PPTs (from baseline to 3 months) was found between groups.
Polaski et al. [48]	Chronic low back pain *N* = 52 F 25 M 27 Age = 37.6 (15.4) years	5 days per wk for 4 wks -Guided meditation recording followed by 30 min of treadmill walking.	Audio-book for 12–17 min followed by a 30-minute rest period 5 times per week for 4 wks	PPT (local & remote) -kg/cm^2^ PPT assessed at participant’s low back (local) and forearms (remote) at specific testing sites using handheld algometer with a 1 cm2 probe (Wagner Instruments, Greenwich, CT, USA). Additional measures: MS, MP, CHI, CHU, CPI, CPU	Pre intervention Post intervention	PPT local Mean (SD) Pre: G1: 5.58(1.8) Post: G1: 5.70(1.6) Pre: G2: 4.71(2.0) Post: G2: 4.78(1.8) PPT remote Pre: G1: 3.63(0.9) Post: G1: 3.93(1.0) Pre: G2: 3.54(1.4) Post: G2: 3.53(1.3) No sig. Rx. effects for constant heat pain intensity, constant heat pain un-pleasantness, pressure pain threshold, constant pressure pain intensity, or constant pressure pain unpleasantness
Holm et al. [45]	Knee Osteoarthritis *N* = 90 F 52 M 38 Age = 64.8 (10.0) years	2 education sessions (1) osteoarthritis disease characteristic, symptoms, risk factors, Rx options. (2) exercise as Rx, coping strategies & self-management Neuromuscular Exercise: twice weekly (60 min sessions) for 12 wks (warm up, circuit exercises, cool down) Strength training: one set of low-intensity, high-repetition (30-60RM) knee extensions followed by four sets of high-intensity (8-12RM) leg-press in gym machines	Neuromuscular exercise(as experimental group) & education (as experimental group)	PPT (local & remote), -kPa PPT; assessed at painful/most painful knee (local) & contralateral knee (remote) using a computer-controlled cuff algometer (Cortex Technology, Hadsund & Aalborg University) including two 13-cm wide cuffs (VBM). TS was assessed by inflating the cuff on the index leg. The participant was then subjected to ten short-lasting pressure stimuli (1-s each), using the previously recorded pain tolerance threshold cuff pressure, with 1-s breaks between each stimulus. CPM; assessed by inflating the cuff on contralateral leg to 70% of recorded PTT as the conditioning stimulus. The cuff on the index leg was inflated continuously with a rate of 1 kPa/s. The participants were instructed to press the pressure release button when the pain was intolerable. TS; assessed by inflating the cuff on the index leg. The participant was subjected to ten short-lasting pressure stimuli (1-s each), using the previously recorded PTT cuff pressure, with 1-s breaks between each stimuli	Pre intervention 6-wks Post intervention	PPT local Marginal means (95% CI) Pre: G1: 22.1(7.9) 6 wks: G1: 23.7(21.4 - 26.0) Post: G1: 24.6(22.1 - 27.1) Pre: G2: 20.4(9.7) 6 wks: G2: 19.7(17.6 - 21.8) Post: G2: 19.6(17.4 - 21.7) PPT remote Pre: G1: 22.9(11.5) 6 wks: G1: 24.7(22.6 - 26.9) Post: G1: 23.4(21.1 - 25.6) Pre: G2: 19.3(8.5) 6 wks: G2: 19.1(17.2 - 21.1) Post: G2: 20.9(18.9 - 22.9) CPM Pre: G1: 1.2(10.3) 6 wks: G1: 2.8(0.1 - 5.4) Post: G1: 3.7(0.9 - 6.5) Pre: G2: 2.3(9.3) 6 wks: G2: 3.8(1.4 - 6.2) Post: G2: 3.3(0.9 - 5.7) TS Pre: G1:1.9(1.4) 6 wks: G1: 1.7(1.3 - 2.2) Post: G1: 1.5(1.1 - 2.0) Pre: G2: 2.3(1.5) 6 wks: G2: 2.1(1.7 - 2.5) Post: G2: 1.5(1.2 - 1.9) Statistically sig. difference between groups, at wk 6 & 12, with higher threshold in experimental group for PPT local. Sig. difference in experimental group at 6 wks but not at 12 wks in PPT remote. No sig. differences between groups in TS or CPM at 6 or 12 wks.
Georgopolulos et al. [51]	Chronic low back pain *N* = 97 F 69 M 28 Age = 57(13) years	CBT based PT	MDT- included workshop sessions delivered by a multidisciplinary team- address chronic pain mechanisms, anatomy, goal-setting techniques, graded exercise & pacing, stress management, challenging negative thoughts, relaxation, imagery & mindfulness as well as communication skills & medication use.	PPT, CPM, TS kPA PPT; assessed using a handheld digital algometer (Medoc-AlgoMed Advanced Medical Systems- Computerised Pressure Algometer, Israel. The brachioradialis muscle, approximately 5 cm distal to the lateral epicondyle, was chosen for all modalities as a site distant from the primary area of pain in individuals with CLBP. TS; assessed twice by repeated application to the forearm of a punctate stimulus (256 mN) using the retractable blunt needle of a specially manufactured pen (MRC Systems GmbH; The Pin Prick, Germany A single punctate stimulus was applied on their dominant forearm, followed by 10 repetitive stimuli at a rate of 1/s. CPM; assessed using contralateral forearm ischaemic pain as the conditioning stimulus, rated as 4 on an 11-point current pain NRS.		Baseline scores only PPT Mean (IQR) 205.8(148.2-297.6) TS 1.0 (0.4–2.8) CPM 59.1(5.6–99.3)
Chimenti et al. [43]	Chronic Achilles tendinopathy *N* = 66 F 37 M 29 Age = 43.4 (15.1) years	3 phase exercise programme: isometric ex, heel raises, spring exercise & Pain Science Education Wk 1–7: 6/7 one-to-one physiotherapy visit. Wk 9-12-instructed to maintain HEP, 10 wks-phone call	Exercise programme (as experimental group) & Pathoanatomical education	PPT (local &remote) kPa PPT; assessed at the Achilles tendon & semitendinosus tendon on painful side (local) & contralateral side (remote) using a pressure algometer (Somedic Algometer TypeII, Horby Sweden, probe 1 cm^2^) CPM; the conditioning stimulus involved participants placing their hand in a cold (6+/-0.5 °C) water bath for 120 s & rating the pain in their hand at 5 s & 20 s. The neutral stimulus involved participants placing their hand in a room temperature (22+/-1.0 °C) water bath for 120 s. A formula was used to calculate CPM. TS technique not described in text.	Pre intervention Post intervention	PPT local Mean(SD) Pre: G1: 476.6 (212.5) Post: G1: 477.0(142.1) Pre: G2: 415.1(171.4) Post: 465.1(242.8) PPT remote Pre: G1: 529.9(202.9) Post: G1: 535.0(214.3) Pre: G2:560.8(262.0) Post: G2: 525.3(242.1) CPM Pre: G1: 27.4(33.6) Post: G1: 14.2(26.1) Pre: G2: 24.4(26.3) Post: G2: 16.9(27.3) TS Pre: G1: 2.1(1.3) Post: G1: 1.9 (1.6) Pre: G2: 2.1(1.4) Post: G2: 2.0(1.2) No sig. changes in PPT local, remote, TS or CPM post intervention
Matias et al. [47]	Chronic idiopathic neck pain *N* = 52 F 43 M 9 Age = 21 (2.0) years	Pain neuroscience education (the neurophysiology of pain, the transition from acute to chronic pain & the nervous system’s ability to modulate the pain experience) & Exercise- aimed at increasing the endurance & strength of the deep neck flexors &extensors 30 min sessions, 1 per wk, 4 wks.	Exercise (as experimental group)	PPT (local) PPT assessed at right & left upper trapezius & the articular pillar of C5/C6 (local), using a pressure algometer (JTECH Medical Industries, Salt Lake City, US)	Pre intervention Post intervention	PPT local Mean (SD) Pre: G1: C1-C2(R) 15.0(5.6) Post: G1: C1-C2(R) 15.4(5.3) Pre: G2: C1-C2(R) 15.0(4.3) Post: G2: C1-C2(R) 16.3(6.0) Pre: G1: C1-C2(L) 14.6(5.7) Post: G1: C1-C2(L) 15.3(5.6) Pre: G2: C1-C2(L) 15.6(4.4) Post: G2: C1-C2(L) 15.9(5.7) Pre: G1: C5-C6® 14.9(5.6) Post: G1: C5-C6® 15.7(6.2) Pre: G2: C5-C6(R) 15.8(4.9) Post: G2: C5-C6(R) 16.1(4.4) Pre: G1: C5-C6(L) 15.3(5.5) Post: G1: C5-C6(L) 15.5(5.6) Pre: G2: C5-C6(L) 16.8(5.6) Post: G2: C5-C6(L) 16.9(5.7) Pre: G1: Mid traps(R) 17.4(5.5) Post: G1: Mid traps(R) 17.5(5.7) Pre: G2: Mid traps(R) 18.4(4.9) Post: G2: Mid traps(R) 17.3(4.7) Pre: G1: Mid traps(L) 17.5(6.0) Post: G1: Mid traps(L) 17.3(5.2) Pre: G2: Mid traps(L) 19.5(5.2) Post:G2: Mid traps(L) 17.4(4.6) Neither intervention nor time was found to have a sig. effect on pressure pain threshold measurements (*P* > 0.05).

Legend

PPT: Pressure pain threshold, CPM: Conditioned pain modulation, TS: Temporal summation, CSI: Central sensitisation inventory, TDT: Temperature detection threshold, TPT: Thermal Pain Threshold, MS: Mechanical sensitivity, MP: Mechanical pain, CHI: Constant heat intensity, CHU: Constant heat unpleasantness, CPI: Constant pressure intensity CPU: Constant pressure unpleasantness, mth: month, &: and, sig: significant, N: number, wk: week, Rx: treatment, G1: experimental group, G2: control group, SD: Standard deviation, SE: Standard Error, IQR: Inter quartile range, CI: Confidence Interval

NOTE: PPT local and remote scores are combined averages in studies where multiple local/remote PPTs were taken

Four trials studied the effect of a combined exercise and psychological intervention on CPM [43, 45, 46, 51]. The study populations in which CPM was performed were chronic low back pain [46], chronic neck pain [51], knee osteoarthritis [45] and chronic Achilles tendinopathy [43]. Two studies utilised cuff inflation on the contralateral limb as the conditioning stimulus [45, 51] and two studies used cold water bath immersion of the contralateral limb as the conditioning stimulus [43, 46].

Three trials studied the effect of a combined exercise and psychological intervention on TS measures [43–45] in the following study populations; knee osteoarthritis [45], chronic neck pain [51] and chronic Achilles tendinopathy [43]. Georgopoulos et al. [51] reported measuring TS using of a single punctate stimulus applied to the dominant forearm using a retractable blunt needle, followed by 10 repetitive stimuli at a rate of 1/s to the forearm. Holm et al. [45] measured TS by utilising an inflating cuff on the index leg. The participant was then subjected to ten short-lasting pressure stimuli (1-s each), using the previously recorded pain tolerance threshold cuff pressure, with 1-s breaks between each stimuli. The TS technique was not described in detail in the study by Chimenti et al. [43]

The CSI scores were recorded in three of the included trials [41, 44, 46] in participants with chronic low back pain [41, 44, 46].

A number of additional QST measures were recorded in the following studies; Polaski et al. [48] measured cutaneous mechanical sensitivity, cutaneous mechanical pain, constant heat pain intensity, constant heat pain unpleasantness, constant pressure pain intensity, constant pressure pain unpleasantness) and Holm et al. [45] measured pain tolerance threshold.

### Interventions

RCT data were included in the meta-analysis (*n* = 10 studies) [41–50] when the trial included an intervention group consisting of a combined exercise and psychological intervention and a comparator group. The psychological elements included in the intervention groups included cognitive functional therapy [53], pain neuroscience education [41, 43, 44, 46, 47], a pain education video [52], individualised psychological support [42], psychologically informed pain education [45, 49, 50], guided meditation [48], CBT [51] and multimodal psychological disability management [53]. The exercise components of the interventions included; exercise of personal preference [53], multimodal home exercise programme including motor control exercises, stretching and aerobic exercise [41], group exercise including strength exercises, motor control and flexibility [52], one-to-one time-contingent exercise programme [46], home exercises featuring ‘five home exercises in five minutes’ [42], supervised exercise programme including neck flexor and extensor function, balance and oculomotor exercise, shoulder girdle exercise [49], group therapeutic exercise [44], neuromuscular exercise [45, 50], treadmill walking [48], progressive heel raise protocol [43] and deep neck flexor and extensor exercise [47]. The time-frame of the treatment intervention period also varied; from four weeks [47, 48], six weeks [44], 12 weeks [43, 46, 50], 16 weeks [41, 49] to approximately 18 weeks [42]. Eight of the combined interventions included in this review were delivered by physiotherapists [41–45, 49, 51, 53]. In addition, a clinical psychologist [48], nurse [49] and an unspecified MDT member [51] were also described as intervention providers. In two of the included studies, the profession of the intervention provider was not specified [46, 52].

Studies compared the experimental group to; a waiting list control [42], exercise only [41, 47], pain education only [49], ‘usual physiotherapy’ [44], a leaflet with general advice [50], listening to an audio-book followed by 30 min resting [48], combined pathoanatomical education and exercise [43], and traditional back school with a biomedical approach combined with a pain contingent exercise programme [46]. One RCT [52] was not included in the meta-analysis as both the experimental group and the comparator group included a combined exercise and psychological intervention.

### Methodological quality

The 10 RCTs were assessed using the PEDRO scale (Table 2). The scores ranged from four to 10 out of a total possible score of 11. The most common biases present was failure to blind the therapist in all 10 included studies and failure to blind the participants in seven out of ten studies. The methodological quality of the remaining three studies was assessed using the Newcastle-Ottawa scale (Tables 3 and 4). This scale has three main criteria; the selection of the study groups, the comparability of the study groups, and the ascertainment of the outcome [32]. The total scores of the included studies ranged from five to seven out of a possible maximum of nine.

**Table 2:** Methodological quality of included randomised controlled trials (PEDro scale)

Reference	1	2	3	4	5	6	7	8	9	10	11	Total
Bodes-Pardo et al. [41]	1	1	1	1	0	0	1	1	1	1	1	9
Malfliet et al. [46]	1	1	1	1	1	0	1	1	1	1	1	10
Cabak et al. [42]	1	1	0	0	0	0	0	0	0	1	1	4
Ris et al. [49]	1	1	1	1	0	0	1	0	1	1	1	8
Galan-Martin et al. [44]	1	1	1	1	0	0	1	1	1	1	1	9
Skou et al. [50]	1	1	1	1	0	0	1	1	1	1	1	9
Polaski et al. [48]	1	1	1	1	0	0	1	0	0	1	1	7
Chimenti et al. [43]	1	1	1	1	1	0	1	1	1	1	1	10
Matias et al. [47]	1	1	1	1	0	0	1	1	0	1	1	8
Holm et al. [45]	1	1	1	1	1	0	1	1	1	1	1	10

Legend

PEDro items: 1 Eligibility criteria; 2 Random allocation; 3 Concealed allocation; 4 Comparability at baseline; 5 Patient blinding; 6 Therapist blinding; 7 Assessor blinding; 8 At least 85% follow-up; 9 Intention to treat analysis; 10 Between-group statistical comparisons; 11 Point measures and measures of variability

**Table 3:** Quality of included cohort studies (Newcastle Ottawa Scale NOS)

Reference	Selection				Comparability	Outcome			
	Representativeness of exposure	Selection of the non- exposed	Ascertainment of exposure	Outcome not present at start of study	Comparability on the basis of design or analysis	Assessment of outcome	Follow-up long enough for outcomes	Adequacy of follow up	Final Score
Sitges et al. [52]	*	*	*	-	-	-	*	*	5
Georgopoulos et al. [51]	*	*	*	-	-	-	*	*	5

Legend

Each item in the table gets a maximum of 1 point, if it meets the evaluation criteria, which is recorded as “*”

“-” denotes that the study did not meet the evaluation criteria

There are 8 evaluation items in total, with a full score of 8 points

A higher score means the least risk of bias

NOS: Newcastle Ottawa Quality Assessment Scale

**Table 4:** Quality of included case-control studies (Newcastle Ottawa Scale NOS)

Reference	Selection				Comparability	Outcome			
	Is the case definition adequate?	Representativeness of the cases	Selection of Controls	Definition of Controls	Comparability on the basis of design or analysis	Ascertainment of exposure	Same method ascertainment for cases and controls	Non-Response rate	Final Score
Vaegter et al. [53]	-	*	*	*	**	*	*	-	7

Legend

Each item in the table gets a maximum of 1 point, if it meets the evaluation criteria, which is recorded as “*”

‘Comparability on the basis of design or analysis’ has a maximum of 2 points available

“-” denotes that the study did not meet the evaluation criteria

There are 8 evaluation items in total, with a full score of 9 points

A higher score means the least risk of bias

NOS: Newcastle Ottawa Quality Assessment Scale

### Meta-analysis

Ten RCTs were included in the meta-analysis [41–50] investigating changes in static QST measures (PPT local, PPT remote), dynamic QST measures (CPM, TS), pain severity and CSI scores.

### PPT local

In terms of local PPT scores, five individual studies [41, 42, 44, 46, 49] showed significant increases in local PPT scores in a combined exercise and psychological intervention group compared to a control group. Four of these studies investigated low back pain [41, 42] or spinal pain [46] and one investigated cervical pain [49]. A further four studies, investigating knee [50], low back [48], Achilles tendon [43] and neck [47] pain, showed no between group differences post intervention.

**Fig. 2:**
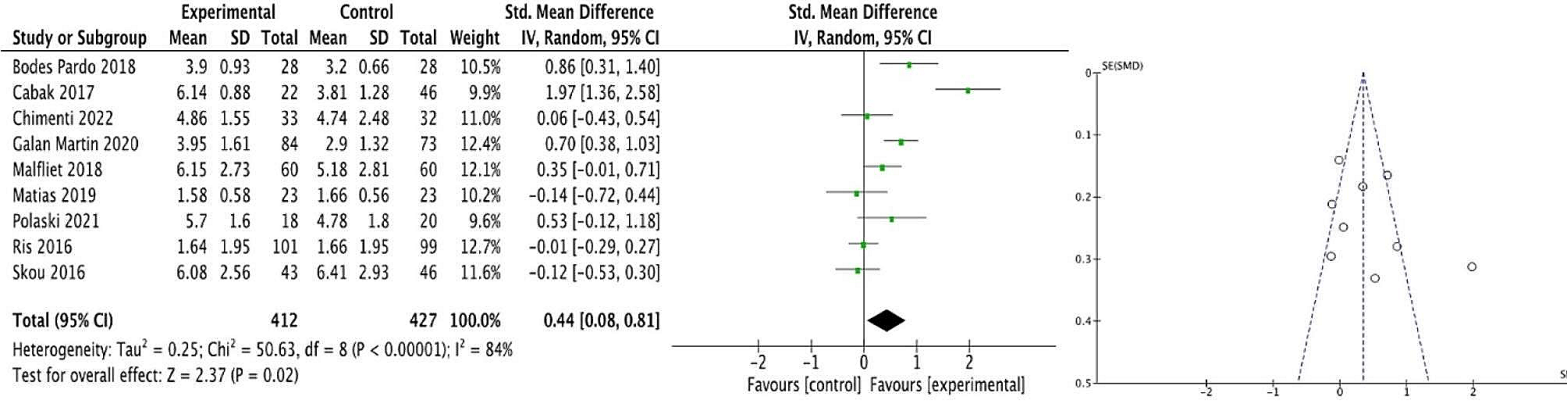
Effect of combined exercise and psychological intervention on post intervention PPT local scores Legend: Forest plot for post-intervention analysis comparing combined exercise and psychological intervention with control group for local PPT scores (left) Funnel plot demonstrating publication bias (right)

Meta-analysis of all nine trials (*n* = 839 participants) post-intervention showed significantly higher PPT scores in favour of the exercise and psychological intervention group, with small effect sizes [SMD = 0.44; 95% CI 0.08–0.81; *P* = 0.02]. The results appear to have high levels of heterogeneity I^2^ = 84%. Visual analysis of the funnel plot demonstrated relative funnel plot symmetry suggesting that there was no significant publication bias (Fig. 2).

### Subgroup analysis (PPT local) according to area of pain

**Fig. 3:**
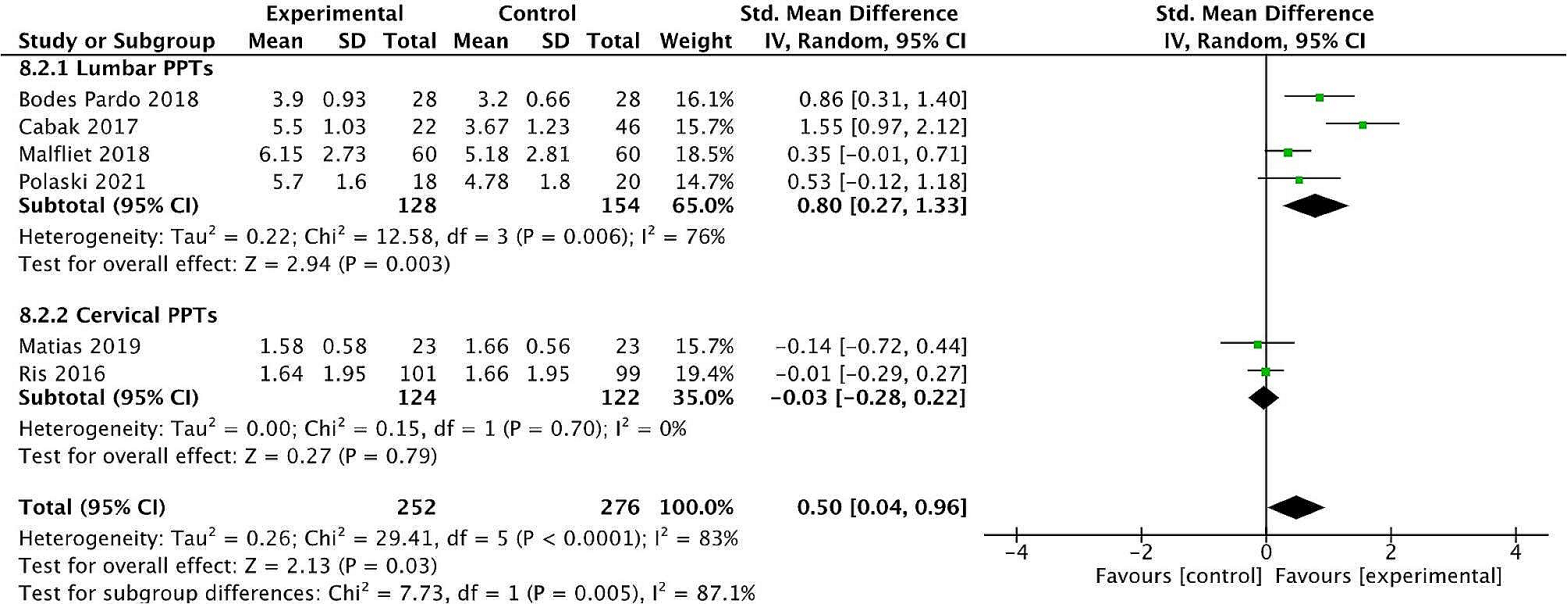
Subgroup analysis of effect of combined exercise and psychological intervention on post intervention PPT local scores Legend: Forest plot for post-intervention analysis comparing combined exercise and psychological intervention with control group for local PPT scores in a subgroup of patients with chronic lumbar and cervical pain

A sub-group analysis of local PPT scores was carried where two or more studies were available, grouping studies according to the primary area of CP of the study participants [41, 42, 46–49]. The areas of CP included were all spinal pain; lumbar spine (*n* = 4 studies) and cervical spine (*n* = 2 studies). For participants with lumbar spine pain, where PPT scores were measured locally in the lumbar spine, the analysis revealed a significantly higher local PPT score in favour of the exercise and psychological intervention group with a large effect size [SMD = 0.80; 95% CI 0.27–1.33; *P* = 0.006] with the results appearing to have high levels of heterogeneity; I^2^ = 76% (Fig. 3). The participants in the sub-group analysis investigating the lumbar spine presented with non-specific chronic low back pain for > 3 months [42, 46] or > 6 months [41, 48]. There were no significant between group differences found in local PPT scores when the primary area of pain was the cervical spine (*n* = 2), with a low effect size [SMD=-0.03; 95% CI 0.28 − 0.22; *P* = 0.79] and the results showing low levels of heterogeneity; I^2^ = 0%.

### PPT remote

In terms of remote PPT scores, one study [49] showed a significant increase in remote PPT scores in the exercise plus psychological intervention group compared to control group post intervention. This study [49] investigated remote PPTs scores in individuals with cervical pain. A further five studies [41, 43, 46, 48, 50] showed no between group differences post intervention. In these five studies, remote PPT scores were investigated in individuals with a number of different pain areas; knee [50], low back [41, 46, 48] and Achilles tendon [43].

**Fig. 4:**
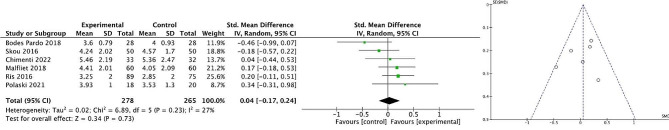
Effect of combined exercise and psychological intervention on post intervention PPT remote scores Legend: Forest plot for post-intervention analysis comparing combined exercise and psychological intervention with control group for remote PPT scores (left) Funnel plot demonstrating publication bias (right)

Meta-analysis of six trials [41, 43, 46, 48–50] (*n* = 543 participants) revealed no significant between group differences in remote PPT scores [SMD = 0.04; 95% CI -0.17-0.24; *P* = 0.73]. The results appear to have low levels of heterogeneity; I^2^ = 27% (Fig. 4). Visual analysis of the funnel plot demonstrated relative funnel plot symmetry suggesting that there was no significant publication bias.

### Conditioned pain modulation

**Fig. 5:**
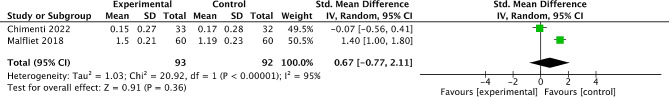
Effect of combined exercise and psychological intervention on post intervention CPM scores Legend: Forest plot for post-intervention analysis comparing combined exercise and psychological intervention with control group for CPM scores

Two studies [43, 46] investigated changes in CPM following a psychological and exercise intervention, in individuals with Achilles tendinopathy [43] and low back pain [46]. Individually neither study found significant between group differences between experimental and control groups post intervention. Meta-analysis of these two trials (*n* = 185 participants) post-intervention revealed significantly higher CPM scores in favour of the control group with a moderate effect size [SMD = 0.67; 95% CI -0.77-2.11; *P* = 0.36]. The results have high levels of heterogeneity I^2^ = 95% (Fig. 5).

### Temporal summation

**Fig. 6:**
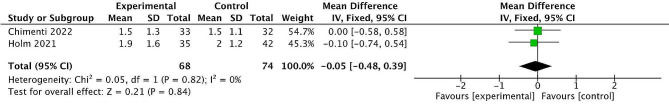
Effect of combined exercise and psychological intervention on post intervention TS scores Legend: Forest plot for post-intervention analysis comparing combined exercise and psychological intervention with control group for TS scores

Two studies [43, 45] investigated changes in TS following a psychological and exercise intervention in individuals with Achilles tendinopathy [43] and knee osteoarthritis [45]. Meta-analysis of the two trials [39, 41] (*n* = 142 participants) post-intervention revealed no significant between group difference in temporal summation scores [SMD=-0.05; 95% CI 0.48 − 0.39; Z = 0.21; *P* = 0.84]. The results have low levels of heterogeneity I^2^ = 0% (Fig. 6).

### Pain intensity outcomes

**Fig. 7:**
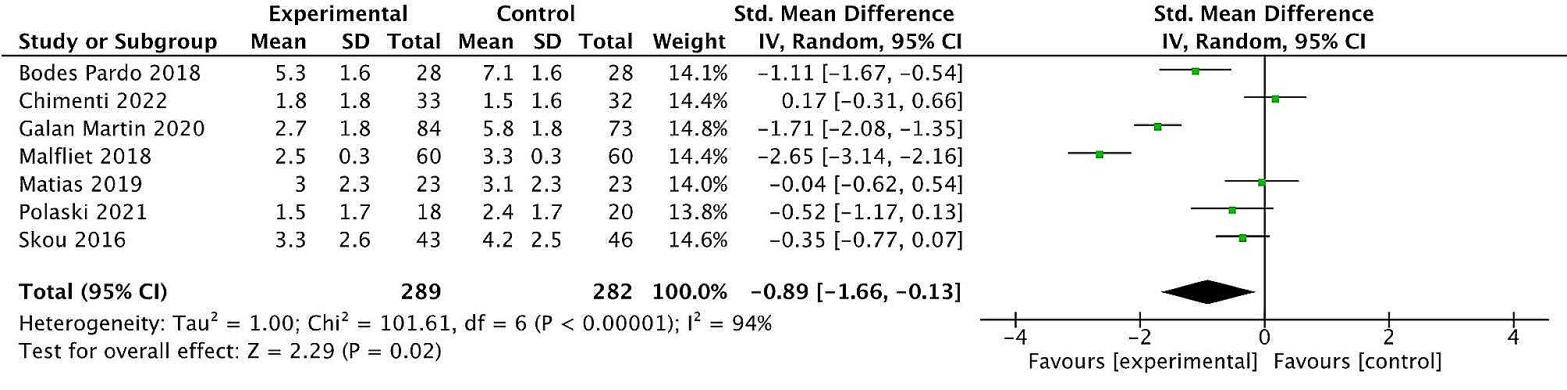
Effect of combined exercise and psychological intervention on post intervention pain intensity scores Legend: Forest plot for post-intervention analysis comparing combined exercise and psychological intervention with control group for pain intensity scores

Seven randomised control trials [41, 43, 44, 46–48, 50] investigated changes in pain scores following a psychological and exercise intervention in individuals with low back pain [41, 46, 48], spinal pain [44], Achilles tendinopathy [43], cervical pain [47] and knee pain [50]. In terms of pain scores, four of these studies showed significant differences between the experimental group and comparator group post intervention [41, 44, 48–50]. Meta-analysis of the seven studies (*n* = 571 participants) demonstrated statistically significant differences in pain intensity were found in favour of the combined exercise and psychological intervention group [SMD=-0.89; 95% CI -1.66- -0.13; *P* = 0.02] with high heterogeneity (I^2^ = 94%) noted in the included studies (Fig. 7).

### Central sensitisation inventory

**Fig. 8:**
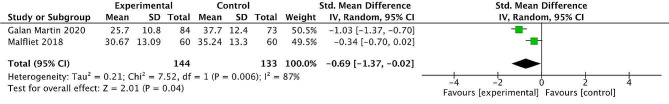
Effect of combined exercise and psychological intervention on post intervention CSI scores Legend: Forest plot for post-intervention analysis comparing combined exercise and psychological intervention with control group for CSI scores

Two RCTs investigated changes in CSI scores following a psychological and exercise intervention in individuals with low back pain [46] and spinal pain [44]. Meta-analysis of the two studies (*n* = 277 participants) showed statistically significant differences in CSI score in favour of the combined exercise and psychological intervention group (*n* = 2 studies), [SMD=-0.69; 95% CI -1.37- -0.02; *P* = 0.006] with high heterogeneity I^2^ = 87% noted in the included studies (Fig. 8). Both studies experimental interventions included pain neuroscience education (PNE), with Malfliet et al. [46] combining PNE with one-on-one exercises sessions over 12 weeks and Galan-Martin et al. [44] combining PNE with group exercise over six weeks.

## Discussion

To our knowledge this is the first systematic review and meta-analysis that has specifically investigated the effects of combined exercise and psychological interventions on measures of nervous system sensitisation in adults with chronic musculoskeletal pain. The current review found combined exercise and psychological interventions compared to a control group demonstrated improvements post-intervention in local PPT scores, patient reported pain scores and CSI scores in adults with chronic musculoskeletal pain and no significant differences in the dynamic QST measures (CPM and TS) or remote PPT scores, It is notable that there was evidence of large heterogeneity in the results for local PPT scores, patient reported pain scores and CSI scores which necessitates caution in interpreting these results.

### PPT local and remote

The most frequently utilised QST measures were local PPTs, investigated in 12 of the included [41–52] studies. In nine [41, 43, 45, 46, 48–52] of those 12 studies, remote PPTs were measured in addition to local PPTs. In the literature, PPT appears to be the most frequently assessed QST parameter [54]. A recent meta-analysis [55] compared PPTs in a pooled sample of individuals with CP (*n* = 1280) and healthy controls (*n* = 1463) and found those with CP had significantly lower PPTs compared with healthy controls (pooled PPT mean difference was − 1.17, 95%CI = − 1.45 to − 0.90).

Meta-analysis of the trials in the current review suggests that following a combined exercise and psychological intervention, local PPTs were significantly increased in adults with mixed chronic musculoskeletal conditions [SMD = 0.44; 95% CI 0.08–0.81; *P* = 0.02] and in a low back pain subgroup [SMD = 0.80; 95% CI 0.27–1.33; *P* = 0.006], when compared to a control intervention. No significant between group differences were found in the remote PPT measures [SMD = 0.04; 95% CI -0.17-0.24; *P* = 0.73]. It is important to observe that high heterogeneity (I^2^) was present in the local PPT score results (I^2^ = 84%). This heterogeneity was anticipated following review of a previous meta-analysis investigating PPT scores [34]. In addition, on investigation of the methodology described in the included studies measuring PPT, and the variable descriptions of the areas measured in both local and remote PPTs, it is notable that variation exists in the testing procedures which may impact the consistency of the results. The random-effects model was utilised in analysis of the data, due to the identified heterogeneity of the studies. As PPTs preferentially activate deep afferent fibres, they are considered an appropriate measure of the local pain areas response to blunt mechanical stimuli, thought to reflect local sensitisation of Aδ and C fibres [56]. Furthermore, when considering remote PPTs, static mechanical allodynia of remote areas assessed by remote PPTs is thought to reflect mechanisms of CS found in nociplastic pain [11]. As CS may be one of the key mechanisms associated with nociplastic pain, the effects of the combined interventions appeared to preferentially affect peripheral pain mechanisms, as measured by hyperalgesia (PPTs). It is notable that a greater number of trials (*n* = 9 studies), with a larger sample size (*n* = 839 participants) were used in the meta-analysis of local PPTs compared to remote PPTs which may contribute to this outcome.

Previous reviews investigating exercise interventions only in individuals with CP have reported some differing results to the current review. A review by Hall et al. [57] investigated the effects of both short- and longer-term exercise interventions on PPTs in individuals with knee osteoarthritis (*n* = 16 studies). Following a single bout of exercise, only local PPTs increased significantly [SMD 0.26, 95% CI 0.02–0.51, *P* = 0.04, I^2^ = 46%]. Following a longer exercise programme (5–12 weeks) no statistically significant changes were found in local [SMD 0.23, 95% CI -0.01-0.47, *P* = 0.06, I^2^ = 64%] or remote PPTs [57]. In the current review, only local PPTs were found to improve following participation in a longer-term (4–18 weeks) exercise intervention combined with a psychological intervention. While no reviews to date have examined the effects of combined exercise and psychological intervention on QST, a recent systematic review [58] investigated the potential benefits of combining exercise with Cognitive Behavioural Therapy (CBT) for adults with chronic diseases. This review [58] highlighted that CBT combined with exercise significantly decreased depression and anxiety with small effect sizes and fatigue with a large effect size, when compared to usual care or waitlist, however no QST measures were reported in the studies included in the review. Considering changes in depression and anxiety may reflect changes in central processing mechanisms, QST measures could be considered as a further investigation of these central nervous system changes.

### CPM and TS

In the current review, CPM was investigated in four of the included studies [43, 45, 46, 51] and TS was investigated in three of the included studies [43, 45, 51]. The meta-analysis of dynamic QST measures in the current review, showed no significant between group differences for TS measures [SMD=-0.05; 95% CI 0.48 − 0.39; *P* = 0.84], and for CPM [SMD = 0.67; 95% CI -0.77-2.11; *P* = 0.36]. Variable outcomes have been shown when CPM has been previously investigated, with reviews reporting mixed results on the CPM effect in pain and control groups [59, 60]. The clinical value of CPM is thought to be in being a measure of the brain’s capacity for activating endogenous analgesia, via the descending tracts [12], which is important to consider in relation to the effects of both exercise and psychological interventions in CP. A number of the psychological interventions of the studies included in the current review included elements of pain neuroscience education [41, 43, 44, 46, 47]. Previous research investigating pain education alone shows that educating individuals with CP about pain mechanisms and challenging maladaptive pain cognitions/behaviours can also alter central pain processing [61, 62]. In individuals with CP, an increased understanding of pain using the “explain pain” [63] concept has been shown to correspond to an improvement in CPM [61].

Due to the small numbers of studies (*n* = 2) included in the meta-analysis investigating CPM in the current review, it is difficult to draw conclusions regarding the findings of the effects of combined exercise and psychological interventions on this QST measure. A number of previous studies investigating TS and CPM focused on exploring the measures as predictors of outcome [64–66]. The studies included in the current meta-analysis utilised these dynamic QST measures as markers of treatment outcome, following participation in combined exercise and psychological interventions. Utilising dynamic QST as markers of treatment outcome has been a more common strategy in studies investigating the central modulatory effects of drug interventions [67, 68], however future research could investigate changes in the central nervous system following participation in combined exercise and psychological interventions.

### Pain and CSI

In the current review, meta-analysis of post intervention pain severity scores improved significantly [SMD=-0.89; CI -1.66- -0.13; *P* = 0.02] in the combined exercise and psychology group compared to the control group for different groups of individuals with CP, i.e. low back pain [41, 46, 48], knee osteoarthritis [50], neck pain [47], spinal pain [44] and Achilles tendinopathy [43]. The average change in mean pain score of the combined studies was 2.7 (SD2.9) in the combined exercise and psychological intervention group and 1.4 (SD2.4) in the control group. This finding is interesting as research highlights that the primary aims of pain management programmes are to reduce emotional distress and improve physical function rather than focusing on eliminating pain [69, 70]. Moreover, for CSI scores, the current meta-analysis showed significant differences post intervention, in favour of the combined exercise and psychological intervention group [SMD=-0.69; CI -1.37- -0.02; *P* = 0.006]. Similar to the findings for local PPT scores, high levels of heterogeneity were found in the CSI score results, and in addition, only two of the studies in this review were included in the meta-analysis. Considering no changes were found in favour of a combined exercise and psychological intervention in the meta-analysis investigating QST measures proposed to investigate the mechanisms involved in central sensitisation (TS, CPM, remote PPT), conflicting results are found in the meta-analysis of the post-intervention CSI scores. This finding may not be unique, as a recent systematic review (*n* = 69 studies) [71] investigated if questionnaires exploring central sensitisation reflected measures of nociceptive sensitisation. In this review the authors investigated the degrees to which the CSI and the Pain Sensitivity Questionnaire assessed nociceptive sensitisation or emotional sensitisation. When the CSI was examined for its correlation with QST measures, the results found no or weak correlations with a number of QST measures; PPTs, TS, and CPM (*r* < 0.3). The CSI was found however to be strongly correlated with psychological measures (anxiety, depression, pain catastrophising, stress, sleep, and kinesiophobia) with the review concluding that the CSI seemed to identify people with a psychological vulnerability that is associated with pain, rather than central sensitisation itself [71]. With the current review examining interventions that were composed in part of psychological components, it may be possible that the changes identified in CSI scores in the meta-analysis, reflected changes in some psychological constructs in the intervention participants. Furthermore, the effects of exercise on psychological outcomes has been outlined in previous studies. A recent review of Cochrane systematic reviews (*n* = 21 systematic reviews) evaluated exercise for individuals with CP [72] highlighting five reviews that assessed psychological function [73–77] and found exercise interventions had significant effects on measures of mental health [75], depression scores [73] and anxiety [77].

In the current meta-analysis, two studies investigated CSI scores. A study investigating the effect of pain neuroscience education combined with cognition-targeted motor control training on chronic spinal pain [46], reported mean baseline scores of 40.02(1.47) were found in the combined exercise and psychology group and 39.88(1.47) in the control group. A second study [44] investigating pain neuroscience education and physical therapeutic exercise for participants with chronic spinal pain, also was found to have high mean CSI baseline scores; 43.4(12.5) in the combined exercise and psychology group and 38.6(11.7) in the control group. The baseline CSI scores in these two studies suggest that the cohorts included in these studies consisted of centrally sensitised individuals, with cut off scores of > 40 on the CSI indicating the presence of CS [78].

### Strengths and limitations

It is a strength of the current review that the majority of included studies examined the QST measure, PPT, allowing a large sample for meta-analysis. In terms of limitations, a smaller number of studies examined CPM, TS and the CSI. The eligibility criteria for patients in this study outlined that studies that investigated individuals with chronic musculoskeletal pain would be included with the classification of chronic musculoskeletal pain guided by the ICD-11 classification for chronic pain. A wide range of different types of musculoskeletal pain conditions were reported in the studies, which may have condition specific implications for the effects of interventions on measures of nervous system sensitisation. However, associations in the heterogeneous sample found in the current review may also point to underlying factors related to pain mechanisms common to multiple chronic musculoskeletal pain conditions. Furthermore, a limitation of this review was the variability found in the comparison groups of the included studies included in the meta-analysis. A large number of studies included in this review used active comparison groups. This is important to consider when combining the effect of the interventions compared to the comparators in this review and is considered a limitation. In addition, it is notable that variation in testing procedures were described in testing PPT, CPM and TS which may have contributed to the heterogeneity found in resulting scores.

## Conclusions

When considering the future of personalised pain treatment, research to date has identified QST measures to be useful to phenotype CP patient subgroups based on different underlying pain mechanisms [79–81], and to predict response to treatment based on mechanistic phenotype [82, 83]. Further research is required to quantify changes in nervous system sensitisation, using QST, following interventions utilising combined exercise and psychological approaches. In order to effectively investigate and pool the data from future research utilising QST measures, it would be helpful if both QST testing procedures and sites of testing were standardised. The ability to quantify change using QST may help measure the effects of such programmes on pain sensitisation measures and facilitate identification of individuals who may benefit from participation in these programmes. Including further longitudinal prospective studies with larger cohorts of individuals with CP in studies to investigate longer term effects of combined exercise and psychological interventions on static and dynamic QST measures could contribute to understanding the effects of this type of intervention on nervous system sensitisation.

### Electronic supplementary material

Below is the link to the electronic supplementary material.

Supplementary Material 1

## Data Availability

The datasets used and/or analysed during the current study are available from the corresponding author on reasonable request.

## Abbreviations

CBT: Cognitive Behavioural Therapy
CI: Confidence Interval
CP: Chronic Pain
CPM: Conditioned Pain Modulation
CS: Central Sensitisation
CSI: Central Sensitisation Inventory
I^2^: Heterogeneity Statistic
PPT: Pressure Pain Threshold
QST: Quantitative Sensory Testing
RCT: Randomised Control Trial
SMD: Standard Mean Difference
TS: Temporal Summation

## References

[1] Banic B, Petersen-Felix S, Andersen OK, Radanov BP, Villiger MP, Arendt-Nielsen L (2004). Evidence for spinal cord hypersensitivity in chronic pain after whiplash injury and in fibromyalgia. Pain.

[2] Daenen L, Nijs J, Raadsen B (2013). Cervical motor dysfunction and its predictive value for long-term recovery in patients with acute whiplash-associated disorders: a systematic review. J Rehabil Med.

[3] Price DD, Staud R, Robinson ME (2002). Enhanced temporal summation of second pain and its central modulation in fibromyalgia patients. Pain.

[4] Jull GA (2015). The neurophysiology of pain and pain modulation: modern pain neuroscience for musculoskeletal physiotherapists.

[5] Chimenti RL, Frey-Law LA, Sluka KA (2018). A mechanism-based approach to physical therapist management of pain. Phys Ther.

[6] Hall T, Briffa K, Schäfer A (2015). Quantitative sensory testing: implications for clinical practice.

[7] Courtney CA, Kavchak AE, Lowry CD, O’Hearn MA (2010). Interpreting joint pain: quantitative sensory testing in Musculoskeletal Management. J Orthop Sports Phys Therapy.

[8] Pavlaković G, Petzke F (2010). The role of quantitative sensory testing in the evaluation of musculoskeletal pain conditions. Curr Rheumatol Rep.

[9] Arendt-Nielsen L, Yarnitsky D (2009). Experimental and clinical applications of quantitative sensory testing applied to skin, muscles and viscera. J Pain.

[10] Pfau DB, Geber C, Birklein F (2012). Quantitative sensory testing of neuropathic pain patients: potential mechanistic and therapeutic implications. Curr Pain Headache Rep.

[11] Kosek E, Clauw D, Nijs J (2021). Chronic nociplastic pain affecting the musculoskeletal system: clinical criteria and grading system. Pain.

[12] Miranda J, Lamana SMS, Dias EV (2015). Effect of pain chronification and chronic pain on an endogenous pain modulation circuit in rats. Neuroscience.

[13] Gatchel RJ, Peng YB, Peters ML (2007). The Biopsychosocial Approach to chronic pain: scientific advances and future directions. Psychol Bull.

[14] Turk DC, Burwinkle TM (2005). Clinical outcomes, cost-effectiveness, and the role of psychology in treatments for chronic pain sufferers. Prof Psychology: Res Pract.

[15] Lumley MA, Schubiner H. Emotional awareness and expression therapy for chronic pain: rationale, principles and techniques, evidence. Critical Review Current Rheumatology Reports. 2019;21:30. 10.1007/s11926-019-0829-6 PMC7309024 31123837

[16] Thiel A, Sudeck G, Gropper H (2020). The IREACT study – a biopsychosocial analysis of the individual response to physical activity. Contemp Clin Trials Commun.

[17] Fransen M, McConnell S, Hernandez-Molina G (2014). Exercise for osteoarthritis of the hip. Cochrane Database Syst Reviews.

[18] Silva KNG, Mizusaki Imoto A, Almeida GJ (2010). Balance training (proprioceptive training) for patients with rheumatoid arthritis. Cochrane Database Syst Reviews.

[19] Busch AJ, Webber SC, Richards RS (2013). Resistance exercise training for fibromyalgia. Cochrane Database Syst Reviews.

[20] Brown J, Brown S (2017). Exercise for Dysmenorrhoea. Cochrane Database Syst Reviews.

[21] Lima LV, Abner TS, Sluka KA (2017). Does exercise increase or decrease pain? Central mechanisms underlying these two phenomena. J Physiol.

[22] Geva N, Defrin R (2013). Enhanced pain modulation among triathletes: a possible explanation for their exceptional capabilities. Pain.

[23] Andrzejewski W, Kassolik K, Brzozowski M (2010). The influence of age and physical activity on the pressure sensitivity of soft tissues of the musculoskeletal system. J Bodyw Mov Ther.

[24] Van Oosterwijck J, Nijs J, Meeus M (2012). Lack of endogenous pain inhibition during exercise in people with chronic whiplash associated disorders: an experimental study. J Pain.

[25] Meeus M, Roussel NA, Truijen S (2010). Reduced pressure pain thresholds in response to exercise in chronic fatigue syndrome but not in chronic low back pain: an experimental study. J Rehabilitation Med.

[26] Higgins JPT, Thomas J, Chandler J, editors. Cochrane handbook for systematic reviews of interventions version 6.3. Cochrane. Available from: www.training.cochrane.org/handbook

[27] Moher D (2009). Preferred reporting items for systematic reviews and meta-analyses: the Prisma statement. Ann Intern Med.

[28] Abenhaim L, Rossignol M, Valat JP (2000). The role of activity in the therapeutic management of back pain. Spine.

[29] Maher CG, Sherrington C, Herbert RD (2003). Reliability of the pedro scale for rating quality of randomized controlled trials. Phys Ther.

[30] Gonzalez GZ, Moseley AM, Maher CG (2018). Methodologic quality and statistical reporting of physical therapy randomized controlled trials relevant to Musculoskeletal conditions. Arch Phys Med Rehabil.

[31] Foley NC, Teasell RW, Bhogal SK (2003). Stroke Rehabilitation evidence-based review: methodology. Top Stroke Rehabil.

[32] Wells GA, Shea B, O’Connell D et al. The Newcastle-Ottawa Scale (NOS) for assessing the quality of nonrandomised studies in meta-analyses. Published January, 2000. Accessed February 2023 http://www.ohri.ca/programs/clinical_epidemiology/oxford.asp

[33] Stang A (2010). Critical evaluation of the Newcastle-Ottawa scale for the assessment of the quality of nonrandomized studies in meta-analyses. Eur J Epidemiol.

[34] Fingleton C, Smart K, Moloney N (2015). Pain sensitization in people with knee osteoarthritis: a systematic review and meta-analysis. Osteoarthr Cartil.

[35] Cumpston M, Li T, Page MJ (2019). Updated guidance for trusted systematic reviews: a new edition of the Cochrane Handbook for systematic reviews of interventions. Cochrane Database Syst Reviews.

[36] Deeks JJ, Higgins JPT, Altman DG. Analysing data and undertaking meta-analyses. Cochrane Handb Syst Reviews Interventions. 2019;241–84.

[37] Huedo-Medina TB, Sánchez-Meca J, Marín-Martínez F (2006). Assessing heterogeneity in meta-analysis: Q statistic or i2 index?. Psychol Methods.

[38] Deeks JJ, Higgins JPT, Altman DG, Higgins J, Thomas J, Chandler J, Cumpston M, Li T, Page M (2019). Analysing data and undertaking meta-analyses. Cochrane Handbook for Systematic Reviews of Interventions.

[39] Takeshima N, Sozu T, Tajika A (2014). Which is more generalizable, powerful and interpretable in meta-analyses, mean difference or standardized mean difference?. BMC Med Res Methodol.

[40] Cohen J (1988). Statistical Power Analysis for the behavioral sciences.

[41] Bodes Pardo G, Lluch Girbés E, Roussel NA (2018). Pain neurophysiology education and therapeutic exercise for patients with chronic low back pain: a single-blind randomized controlled trial. Arch Phys Med Rehabil.

[42] Cabak A, Rudnicka A, Kulej L (2017). Biopsychosocial Rehabilitation Programme for patients with chronic back pain. Pilot study. Ortop Traumatologia Rehabilitacja.

[43] Chimenti RL, Post AA, Rio EK (2022). The effects of pain science education plus exercise on pain and function in chronic achilles tendinopathy: a blinded, placebo-controlled, explanatory, randomized trial. Pain.

[44] Galan-Martin MA, Montero-Cuadrado F, Lluch-Girbes E (2020). Pain neuroscience education and physical therapeutic exercise for patients with chronic spinal pain in Spanish physiotherapy primary care: a pragmatic randomized controlled trial. J Clin Med.

[45] Holm PM, Petersen KK, Wernbom M (2021). Strength training in addition to neuromuscular exercise and education in individuals with knee osteoarthritis—the effects on pain and sensitization. Eur J Pain.

[46] Malfliet A, Kregel J, Coppieters I (2018). Effect of pain neuroscience education combined with cognition-targeted motor control training on chronic spinal pain. JAMA Neurol.

[47] Matias BA, Vieira I, Pereira A (2019). Pain neuroscience education plus exercise compared with exercise in university students with chronic idiopathic neck pain. Int J Therapy Rehabilitation.

[48] Polaski AM, Phelps AL, Smith TJ (2021). Integrated meditation and exercise therapy: a randomized controlled pilot of a combined nonpharmacological intervention focused on reducing disability and pain in patients with chronic low back pain. Pain Med.

[49] Ris I, Søgaard K, Gram B (2016). Does a combination of physical training, specific exercises and pain education improve health-related quality of life in patients with chronic neck pain? A randomised control trial with a 4-month follow up. Man Therap.

[50] Skou ST, Roos EM, Simonsen O (2016). The efficacy of non-surgical treatment on pain and sensitization in patients with knee osteoarthritis: a pre-defined ancillary analysis from a randomized controlled trial. Osteoarthr Cartil.

[51] Georgopoulos V, Akin-Akinyosoye K, Smith S (2022). An observational study of centrally facilitated pain in individuals with chronic low back pain. PAIN Rep.

[52] Sitges C, Terrasa JL, García-Dopico N (2022). An Educational and Exercise Mobile phone-based intervention to elicit electrophysiological changes and to improve psychological functioning in adults with nonspecific chronic low back Pain (BackFit App): Nonrandomized Clinical Trial. JMIR.

[53] Vaegter HB, Ussing K, Johansen JV (2019). Improvements in clinical pain and experimental pain sensitivity after cognitive functional therapy in patients with severe persistent low back pain. PAIN Rep.

[54] Amiri M, Alavinia M, Singh M (2020). Pressure Pain threshold in patients with Chronic Pain: a systematic review and Meta-analysis. Am J Phys Med Rehabil.

[55] Suokas AK, Walsh DA, McWilliams DF (2012). Quantitative sensory testing in painful osteoarthritis: a systematic review and meta-analysis. Osteoarthr Cartil.

[56] Treede R-D, Rolke R, Andrews K (2002). Pain elicited by blunt pressure: neurobiological basis and clinical relevance. Pain.

[57] Hall M, Dobson F, Plinsinga M (2020). Effect of exercise on pain processing and motor output in people with knee osteoarthritis: a systematic review and meta-analysis. Osteoarthr Cartil.

[58] Bernard P, Romain A-J, Caudroit J (2018). Cognitive behavior therapy combined with exercise for adults with chronic diseases: systematic review and meta-analysis. Health Psychol.

[59] O’Brien AT, El-Hagrassy MM, Rafferty H (2019). Impact of therapeutic interventions on pain intensity and endogenous pain modulation in knee osteoarthritis: a systematic review and meta-analysis. Pain Med.

[60] Neelapala YV, Bhagat M, Frey-Law L (2019). Conditioned pain modulation in chronic low back pain. Clin J Pain.

[61] Van Oosterwijck J, Meeus M, Paul L (2013). Pain physiology education improves health status and endogenous pain inhibition in fibromyalgia. Clin J Pain.

[62] Archer KR, Motzny N, Abraham CM (2013). Cognitive-behavioral–based physical therapy to improve surgical spine outcomes: a case series. Phys Ther.

[63] Moseley GL, Butler DS (2017). Explain Pain Supercharged.

[64] Jull G, Kenardy J, Hendrikz J (2013). Management of acute whiplash: a randomized controlled trial of multidisciplinary stratified treatments. Pain.

[65] Coronado R, Bialosky J, Bishop M (2015). The comparative effects of spinal and peripheral thrust manipulation and exercise on pain sensitivity and the relation to clinical outcome: a mechanistic trial using a shoulder pain model. J Orthop Sports Phys Ther.

[66] Henriksen M, Klokker L, Graven-Nielsen T (2014). Association of exercise therapy and reduction of pain sensitivity in patients with knee osteoarthritis: a randomized controlled trial. Arthritis Care Res (Hoboken).

[67] Niesters M, Aarts L, Sarton E (2013). Influence of ketamine and morphine on descending pain modulation in chronic pain patients: a randomized placebo-controlled cross-over proof-of-concept study. Br J Anaesth.

[68] Yarnitsky D, Granot M, Nahman-Averbuch (2012). Conditioned pain modulation predicts duloxetine efficacy in painful diabetic neuropathy. Pain.

[69] Flor H, Fydrich T, Turk DC (1992). Efficacy of multidisciplinary pain treatment centers: a meta-analytic review. Pain.

[70] Hoffman BM, Papas RK, Chatkoff DK (2007). Meta-analysis of psychological interventions for chronic low back pain. Health Psychol.

[71] Adams GR, Gandhi W, Harrison R (2022). Do ‘central sensitization’ questionnaires reflect measures of nociceptive sensitization or psychological constructs? A systematic review and meta-analyses. Pain.

[72] Geneen LJ, Moore RA, Clarke C (2017). Physical activity and exercise for chronic pain in adults: an overview of Cochrane reviews. Cochrane Database Syst Reviews.

[73] Busch AJ, Webber SC, Richards RS (2013). Resistance exercise training for fibromyalgia. Cochrane Database Syst Reviews.

[74] Boldt I, Eriks-Hoogland I, Brinkhof MWG (2014). Non-pharmacological interventions for chronic pain in people with spinal cord injury. Cochrane Database Syst Reviews.

[75] Bartels EM, Lund H, Hagen KB (2016). Aquatic exercise for the treatment of knee and hip osteoarthritis. Cochrane Database Syst Reviews.

[76] Bidonde J, Busch AJ, Webber SC (2014). Aquatic exercise training for fibromyalgia. Cochrane Database Syst Reviews.

[77] Cramp F, Hewlett S, Almeida C (2013). Non-pharmacological interventions for fatigue in rheumatoid arthritis. Cochrane Database Syst Reviews.

[78] Neblett R, Cohen H, Choi YH (2013). The Central Sensitization Inventory (CSI): establishing clinically significant values for identifying central sensitivity syndromes in an outpatient chronic pain sample. J Pain.

[79] Tanaka K, Murata S, Nishigami T (2019). The Central Sensitization Inventory predicts pain-related disability for musculoskeletal disorders in the primary care setting. Eur J Pain.

[80] Phillips K, Clauw DJ (2011). Central pain mechanisms in chronic pain states – maybe it is all in their head. Best Pract Res Clin Rheumatol.

[81] van Wijk G, Veldhuijzen DS (2010). Perspective on diffuse noxious inhibitory controls as a model of endogenous pain modulation in clinical pain syndromes. J Pain.

[82] Staud R (2012). Abnormal endogenous pain modulation is a shared characteristic of many chronic pain conditions. Expert Rev Neurother.

[83] Eisenberg E, Midbari A, Haddad M (2010). Predicting the analgesic effect to oxycodone by ‘static’ and ‘dynamic’ quantitative sensory testing in healthy subjects. Pain.

