# Supplementary material for: The effectiveness of a combined exercise and psychological treatment programme on measures of nervous system sensitisation in adults with chronic musculoskeletal pain - a systematic review and meta-analysis
Source: BMC Musculoskelet Disord. 2024 Feb 14;25:140. doi: 10.1186/s12891-024-07274-8 (PMC10865570; doi:10.1186/s12891-024-07274-8)
Supplement: Supplementary file 1 — Supplementary Material 1 [file 12891_2024_7274_MOESM1_ESM.docx]

**S1: Search Strategy example**

**Search Strategy for Medline Database**

| Population | Intervention | Measurement tool |
| --- | --- | --- |
| "Low Back Pain"[Mesh] OR "Back Pain"[Mesh] OR "sciatica"[Mesh] OR "Failed Back Surgery Syndrome" [Mesh] OR "Spondylosis"[Mesh] OR “low back pain” OR “back pain” OR “sciatica” OR “failed back surgery syndrome” OR “spondylosis” OR Lumbago OR "back ache" OR "backache" OR "Vertebrogenic Pain Syndrome" OR "back disorder" or "lumbar spine pain" OR "sciatic neuralgia" OR "dorsalgia" OR "Intervertebral Disc Degeneration"[Mesh] OR "Scoliosis"[Mesh] OR "Scoliosis" OR "Intervertebral Disc Degeneration” OR "disc degradation" OR "degenerative intervertebral disc" OR "degenerative intervertebral disk" OR "disc degeneration" OR "disk degeneration" OR "lumbarsacral spondylosis" OR “lumbal pain” OR “coccydynia” OR "intervertebral disc injury" OR "lumbosacroiliac pain" OR "intervertebral disk injury" OR "loin pain" OR "lumbosacral pain" OR "discogenic pain" OR "disc herniation" OR "disk herniation" OR "ischialgia" OR "intervertebral disc disease" OR "intervertebral disk disease" OR "lumbar pain" OR "zygapophyseal joint" OR "myofascial pain syndrome" OR “spondylarthritis” OR "spinal fusion" OR "spondylolisthesis" OR "discitis" OR "intervertebral disc displacement" OR "intervertebral disk displacement" OR “vertebral canal stenosis” OR "piriformis muscle syndrome" OR "spinal disease" OR "spinal osteochondrosis" OR "spinal stenosis" OR "spondylitis" OR "discectomy" OR "Neck Pain"[Mesh] OR "Neck Pain" OR “Neck” OR "cervical pain" OR "Whiplash Injuries" OR "whiplash" OR "Facial Pain"[Mesh] OR "Facial Pain" OR "jaw pain" OR “spinal osteophystosis” OR “shoulder pain” [Mesh] OR "Shoulder Pain" OR “shoulder impingement syndrome” [Mesh] OR “shoulder impingement syndrome” OR “shoulder impingement” OR “scapular pain” OR "arm pain" OR "Elbow Pain" OR "Tennis Elbow” [Mesh] OR "tennis elbow” OR "elbow tendinopathy” [Mesh] OR “elbow tendinopathy” OR "Golf Elbow" OR "golfers elbow" OR "wrist pain" OR "hand pain" OR “finger pain” OR "upper limb pain" OR “carpometacarpal joint” OR “thumb pain” OR “metacarpal pain” OR "thoracic pain" OR “thoracalgia” OR “rib pain” OR "chondritis" OR "Pelvic Pain"[Mesh] OR "Pelvic Pain" OR "hip pain" OR "thigh pain" OR "knee pain" OR "ankle pain" OR "foot pain" OR "toe pain" OR "heel pain" OR "leg pain" OR "lower limb pain" OR “patellofemoral pain syndrome” [Mesh] OR “Patellofemoral Pain Syndrome" OR “achilles tendon” OR “patella” OR “groin pain” OR "joint pain" OR "Muscle Pain" OR "muscle pain" OR "Myalgia"[Mesh] OR "myalgia" OR "Arthritis"[Mesh] OR "Arthritis" OR "Arthralgia"[Mesh] OR “arthralgia” OR “arthropathy” OR “arthrochondritis" OR “arthrosynovitis” OR “Tendinopathy"[Mesh] OR "Tendinopathy" OR "tendonitis" OR "tenosynovitis" OR "bone pain" OR "ligament pain" OR bursa OR "Bursitis"[Mesh] OR "bursitis" OR "Capsulitis " OR "synovitis" OR "myofascial pain" OR "impingement" OR "Fasciitis” [Mesh] OR "fasciitis" OR "musculoskeletal pain” [Mesh] OR "musculoskeletal pain” OR “Widespread pain” OR “osteoarthritis” [Mesh] OR “osteoarthritis” OR “osteoarthrosis” OR “spine fusion” OR “spine disease” OR “lumbodynia” OR “periarthritis” | "physical therapy modalities"[mesh] OR "physiotherapy" OR "physical therapy" OR "musculoskeletal manipulation*"[mesh] OR "manual therapy" OR "massage"[mesh] OR "manipulative therapy" OR "myofascial release" OR “education” OR "cognitive behavioral therapy"[mesh] OR "cognitive therapy" OR "cognitive psychotherapy" OR "cognition therapy" OR “mindfulness” OR "acceptance and commitment therapy" OR "resistance training"[mesh] OR strength* OR "trigger point release" OR "dry needling"[mesh] OR "exercise"[mesh] OR "neuroscience education" OR "neurodynamics" OR "stretching" OR "pilates" OR "tai chi" OR "kinesiology, applied"[mesh] OR "hydrotherapy"[mesh] OR "multidisciplinary pain program*" OR "back school" OR "multimodal rehabilitation" OR "multidisciplinary rehabilitation" OR "acupressure" OR "bodywork" OR "soft tissue therapy" OR "manipulative medicine" OR "functional training" OR "aquatic therapy" | "quantitative sensory testing" OR pressure pain threshold* OR " temporal summation" OR "conditioned pain modulation" OR "central sensitisation inventory" OR "central sensitization inventory" OR "thermal stimulus" OR "fibromyalgia impact questionnaire" OR "mechanical pain sensitivity" OR "allodynia" OR "detection threshold" OR "pain sensitivity questionnaire" OR "central pain modulation" OR "Neural Inhibition"[Mesh] OR "Pain Threshold"[Mesh] OR "allodynia" OR "Hyperalgesia"[Mesh] OR "Central Nervous System Sensitization"[Mesh] OR "Nociception"[Mesh] OR nociperception OR hypersensitivity OR "pain perception" OR "sensory threshold" OR "Hyperesthesia"[Mesh] OR "pain sensitization" OR "pain sensitisation" OR "Central Sensitization" OR "Central Sensitisation" OR "peripheral sensitisation" OR "peripheral sensitization" "central hyperexcitability" OR "pain modulation" OR "algometry" OR "heat pain" OR "cold pain" OR "pain amplification" OR "psychophysical testing"  NOT (“mouse” OR “mice” OR “rat”) |

**Search Strategy for Cinahl Database**

| Population | Intervention | Measurement tool |
| --- | --- | --- |
| ((MM "Neck Pain") OR "neck pain" OR "cervical pain" OR (MH "Whiplash Injuries") OR "whiplash" OR (MH "Facial Pain") OR "Facial Pain" OR "Face Pain" OR "jaw pain" OR (MH "Shoulder Pain") OR "Shoulder Pain " OR “scapular pain” OR "arm pain" OR (MH "Elbow Pain") OR "elbow pain" OR (MH "Tennis Elbow") OR “tennis elbow” OR "epicondyl*" OR (MH "Golf Elbow") OR "golfers elbow" OR "wrist pain" OR "hand pain" OR “finger pain” OR “carpometacarpal joint” OR “metacarpal pain” OR "thora* pain" OR “thoracalgia” OR “rib pain” OR "chondritis" OR (MM "Low Back Pain") OR (MM "Back Pain+") OR (MH "Sciatica") OR (MH "Failed Back Surgery Syndrome") OR (MH "Piriformis Syndrome") OR (MH "Intervertebral Disk Displacement") OR (MH "Spondylarthritis") OR (MH "Spondylosis+") OR (MH "Coccydynia") OR (MH "Myofascial Pain Syndromes") OR (MH "Sacroiliac Joint Dysfunction") OR "Sacroiliac Joint Dysfunction" OR "low back pain" OR "back pain" OR "backpain" OR "sciatica" OR "failed back surgery syndrome" OR "piriformis syndrome" OR “discogenic pain” OR "Intervertebral Displacement" OR "Spondylosis" OR "Coccydynia" OR "Myofascial Pain Syndrome" OR "lumbago" OR "lower back pain" OR "back ache" OR "backache" OR "Vertebrogenic Pain Syndrome" OR "back disorder" or "lumbar spine pain" OR "sciatic neuralgia" OR "dorsalgia" OR "sciatic neuropathy" OR "dis* degradation" OR "degenerative intervertebral dis*" OR "dis* degeneration" OR "intervertebral dis* injury" OR "loin pain" OR "lumbosacral pain" OR "dis*ogenic pain" OR "dis* herniation" OR "ischialgia" OR "intervertebral dis* disease" OR "lumbar pain" OR "spinal fusion" OR "spondylolisthesis" OR "dis*itis" OR "spinal disease" OR "spinal osteochondrosis" OR "spine disease" OR "spinal osteophytosis" OR "spondylolysis" OR "spinal stenosis" OR "spondylitis" OR "dis*ectomy" OR "spondylarthritis" OR "lumbal pain" OR "lumbodynia" OR "lumbosacroiliac pain" OR "intervertebral dis* hernia" OR "intervertebral dis* degeneration" OR "posture disorder" OR "spine abnormality" OR "kyphosis" OR "scoliosis" OR "spondylodis*itis" OR "sacrocoxalgia" OR "vertebral canal stenosis" OR (MH "Pelvic Pain") OR "pelvi* pain" OR "hip pain" OR "thigh pain" OR (MH "Knee Pain") OR "knee pain" OR "ankle pain" OR "foot pain" OR "toe pain" OR "heel pain" OR "foot pain" OR "joint pain" OR (MH "Muscle Pain") OR "muscle pain" OR "myalgia" OR "upper limb pain" OR "lower limb pain" OR "leg pain" OR (MH "Arthritis+") OR "arthritis" OR "arthralgia" OR “arthropathy” OR “ arthrochondritis” OR “arthrosynovitis” OR (MH "Tendinopathy") OR "tendinopathy" OR "tendonitis" OR "tenosynovitis" OR "bone pain" OR "ligament pain" OR (MH "Bursitis+") OR "bursitis" OR "capsulitis" OR (MH "Synovitis") OR "synovitis" OR (MH "Myofascial Pain Syndromes") OR "myofascial pain" OR "impingement" OR (MH "Patellofemoral Pain Syndrome") OR "Patellofemoral Pain" OR (MH "Fasciitis+") OR "fasciitis" OR "musculoskeletal pain" OR “Widespread pain” OR “spine fusion” OR “spine disease” OR “periarthritis”) | ((MH "Physical Therapy+") OR (MM "Pain Management") OR (MH "Rehabilitation+") OR (MH "Cognitive Therapy+") OR (MH "Behavior Therapy") OR (MH "Resistance Training") OR (MH "Muscle Strengthening+") OR (MH "Therapeutic Exercise+") OR (MH "Kinesiology") OR (MH "Hydrotherapy") OR (MH "Functional Training") OR "Physical Therapy" OR "Pain Management" OR "back exercise" OR "endurance training" OR "Cognitive Therapy" OR "Cognitive Psychotherapy" OR "Cognitive Behavio*ral therapy" OR "CBT" OR "Movement therapy" OR "Neuropsychological rehabilitation" OR "Behavio*r Therapy" OR "Resistance Training" OR "Muscle Strengthening" OR "Therapeutic Exercise*" OR "Kinesiology" OR "Hydrotherapy" OR "Functional Training" OR "physiotherapy" OR "medical rehabilitation" OR "education" OR "cognition therapy" OR "mindfulness" OR "acceptance and commitment therapy" OR "neurodynamics" OR "muscle stretching" OR "pilates" OR "tai chi" OR "aquatic therapy" OR "aquatic exercise" OR "physical training" OR "weight training" OR "multidisciplinary pain program*" OR "back school" OR "Rehabilitation") | ((MH "Allodynia") OR (MH "Hyperesthesia") OR (MH "Pain Threshold") OR "Allodynia" OR "Hyperesthesia" OR "Pain Threshold" OR "quantitative sensory testing" OR " pressure pain threshold" OR "PPT" OR "QST' OR "temporal summation" OR "conditioned pain modulation" OR "central sensiti*ation inventory" OR "punctate sharpness threshold" OR "mechanical pain sensitivity" OR "detection threshold" OR "pain sensitivity questionnaire" OR "central pain modulation" OR "Neural Inhibition" OR "Central Nervous System Sensiti*ation" OR pain perception" OR "sensory threshold" OR "oxyesthesia" OR "pain sensiti*ation" OR "Central Sensiti*ation" OR "peripheral sensiti*ation" OR "algometry" OR "heat pain" OR "cold pain" OR "pain amplification" OR "psychophysical testing" or "cold hyperalgesia" or "heat hyperalgesia" OR "vibration detection" OR "thermal hyperalgesia" OR "static light touch" OR "blunt pressure" OR "pinprick" OR "nociceptive flexion reflex" OR "sensation detection threshold" OR "spatial summation") NOT(“mouse” OR “mice” OR “rat”) |

**Search Strategy for Embase Database**

| **Population** | **Intervention** | **Measurement tool** |
| --- | --- | --- |
| ('low back pain'/exp OR 'backache'/exp OR 'intervertebral disk disease'/exp OR 'intervertebral disk hernia'/exp OR 'spine fusion'/exp OR 'discectomy'/exp OR 'spine disease'/exp OR 'vertebral canal stenosis'/exp OR 'spondylolisthesis'/exp OR 'spondylitis'/exp OR 'scoliosis'/exp OR 'intervertebral disk degeneration'/de OR 'coccydynia' OR 'sciatica'/de OR 'piriformis syndrome'/de OR 'failed back surgery syndrome'/de OR 'discogenic pain'/de OR 'ischialgia'/de OR 'diskitis'/de OR 'spondylosis'/de OR 'kyphosis'/de OR 'kyphosis' OR 'low back pain' OR 'backache' OR 'back ache' OR 'lumbago' OR 'intervertebral dis* disease' OR 'scoliosis' OR 'spine fusion' OR 'discectomy' OR 'intervertebral dis* degeneration' OR 'dorsalgia' OR 'lumbar pain' OR 'back pain' OR 'backpain' OR 'sacrocoxalgia' OR 'posture disorder' OR 'spine abnormality' OR 'sciatica' OR 'spondylosis' OR 'back disorder' OR 'spondylodis*itis' OR 'myofascial pain syndrome' OR 'vertebrogenic pain syndrome' OR 'failed back surgery' OR 'sciatic neuralgia' OR 'sciatic neuropathy' OR 'discogenic pain' OR 'lower back pain' OR 'dis* degeneration' OR 'dis* degradation' OR 'degenerative intervertebral dis*' OR 'spondylolysis' OR 'lumbar spine pain' OR 'intervertebral dis* hernia' OR 'dis* herniation' OR 'loin pain' OR 'lumbosacral pain' OR 'dis*ogenic pain' OR 'ischialgia' OR 'intervertebral dis* injury' OR 'spinal fusion' OR 'spondylolisthesis' OR 'dis*itis' OR 'intervertebral dis* displacement' OR 'piriformis syndrome' OR 'spin* disease' OR 'spinal osteochondrosis' OR 'spinal osteophytosis' OR 'vertebral canal stenosis' OR 'spinal stenosis' OR 'spondylitis' OR 'dis*ectomy' OR 'lumbosacroiliac pain' OR 'lumbodynia' OR 'lumbal pain' OR 'spondylarthritis' OR 'sacroiliac joint dysfunction' OR 'neck pain'/exp OR 'neck pain' OR 'cervical pain' OR 'whiplash' OR 'face pain'/mj OR 'facial pain' OR 'jaw pain' OR 'shoulder pain'/exp OR 'shoulder pain' OR 'scapular pain' OR 'arm pain'/exp OR 'arm pain' OR 'elbow pain' OR 'tennis elbow'/exp OR 'tennis elbow' OR 'epicondyl*' OR 'golfers elbow' OR 'wrist pain'/exp OR 'wrist pain' OR 'hand pain'/exp OR 'hand pain' OR 'finger pain' OR 'carpometacarpal joint' OR 'metacarpal joint' OR 'thora* pain' OR 'thoracalgia' OR 'thorax pain'/exp OR 'rib pain' OR 'chondritis'/exp OR 'chondritis' OR 'pelvic pain'/exp OR 'pelvi* pain' OR 'hip pain'/exp OR 'hip pain' OR 'thigh pain' OR 'knee pain'/exp OR 'knee pain' OR 'ankle pain'/exp OR 'foot pain'/exp OR 'toe pain' OR 'foot pain' OR 'talalgia' OR 'heel pain'/exp OR 'heel pain' OR 'joint pain' OR 'arthralgia'/exp OR 'myalgia'/exp OR 'myalgia' OR 'muscle pain' OR 'upper limb pain' OR 'lower limb pain' OR 'leg pain' OR 'arthrochondritis' OR 'arthrosynovitis' OR 'arthritis'/exp OR 'arthritis' OR 'arthralgia' OR 'tendinitis'/exp OR 'tendinitis' OR 'tendinopathy' OR 'tenosynovitis' OR 'bone pain' OR 'ligament pain' OR 'arthropathy' OR 'bursitis'/exp OR 'bursitis' OR 'capsulitis' OR 'periarthritis' OR 'synovitis'/exp OR 'synovitis' OR 'myofascial pain'/exp OR 'myofascial pain' OR 'impingement' OR 'patellofemoral pain syndrome'/exp OR 'patellofemoral pain' OR 'fasciitis' OR 'musculoskeletal pain' OR 'widespread pain') | ('physiotherapy'/exp OR 'manipulative medicine'/exp OR 'musculoskeletal manipulation'/exp OR 'massage'/exp OR 'dry needling'/exp OR 'kinesiology'/exp OR 'cognitive behavioral therapy'/exp OR 'resistance training'/exp OR 'movement therapy'/exp OR 'muscle stretching'/de OR 'hydrotherapy'/de OR 'education'/de OR 'myofascial release' OR 'education' OR 'pain management' OR 'endurance training' OR 'cognitive psychotherapy' OR 'therapeutic exercise' OR 'muscle strengthening' OR 'trigger point release' OR 'neurodynamics' OR 'medical rehabilitation' OR 'behavio*r therapy' OR 'cognition therapy' OR 'weight training' OR 'muscle stretching' OR 'pilates' OR 'tai chi' OR 'hydrotherapy' OR 'multidisciplinary pain program*' OR 'back school' OR 'back exercises' OR 'movement therapy' OR 'physical training' OR 'acceptance and commitment therapy' OR 'mindfulness' OR 'acupressure' OR 'bodywork' OR 'soft tissue therapy' OR 'shockwave therapy' OR 'functional training' OR 'aquatic therapy' OR 'neuropsychological rehabilitation' OR 'aquatic exercise' OR 'physiotherapy' OR 'manipulative therapy' OR 'manipulative medicine' OR 'musculoskeletal manipulation' OR 'physical therapy' OR 'massage' OR 'manual therapy' OR 'cognitive behavio*ral therapy' OR 'cbt' OR 'cognitive therapy' OR 'resistance training' OR 'dry needling' OR 'kinesiology' OR 'rehabilitation' OR 'manipulation') | ('allodynia'/exp OR 'quantitative sensory testing'/de OR 'pressure pain threshold'/de OR 'pain threshold'/exp OR 'cold hyperalgesia'/de OR 'heat hyperalgesia'/de OR 'ppt' OR 'qst' OR 'temporal summation' OR 'conditioned pain modulation' OR 'central sensiti*ation inventory' OR 'punctate sharpness threshold' OR 'mechanical pain sensitivity' OR 'detection threshold' OR 'central pain modulation' OR 'neural inhibition' OR 'central nervous system sensiti*ation' OR 'pain perception' OR 'sensory threshold' OR hyperesthesia OR oxyesthesia OR 'pain sensiti*ation' OR 'central sensiti*ation' OR 'peripheral sensiti*ation' OR 'heat pain' OR 'cold pain' OR algometry OR 'pain amplification' OR 'psychophysical testing' OR 'quantitative sensory testing' OR 'pressure pain threshold' OR 'pain threshold' OR 'cold hyperalgesia' OR 'heat hyperalgesia' OR 'allodynia' OR 'vibration detection' OR 'thermal hyperalgesia' OR 'static light touch' OR 'blunt pressure' OR 'pinprick' OR 'nociceptive flexion reflex' OR 'sensation detection threshold' OR 'spatial summation') NOT ('mouse' OR 'mice' OR 'rat') |

**Search Strategy for SPORTDiscus Database**

| **Population** | **Intervention** | **Measurement tool** |
| --- | --- | --- |
| (DE "NECK pain" OR "NECK pain" OR “cervical pain” OR DE "WHIPLASH injuries" OR "WHIPLASH injuries" OR “whiplash” OR DE "FACIAL pain" OR "FACIAL pain" OR “face pain” OR “jaw pain” OR DE "SHOULDER pain" OR "SHOULDER pain" OR “scapular pain” OR “arm pain” OR (DE "ELBOW pain") OR “elbow pain” OR DE "TENNIS elbow" OR “tennis elbow” OR DE "BURSITIS" OR “bursitis” OR DE "ELBOW injuries" OR “epicondylitis” OR (DE "GOLF elbow") OR “golf elbow” OR “golfers elbow” OR “Wrist pain” OR “hand pain” OR “finger pain” OR “carpometacarpal joint” OR “metacarpal pain” OR “thoracic pain” OR “thoracalgia” OR “chondritis” OR DE "LUMBAR pain" OR “lumbar pain OR “low back pain” OR DE "BACKACHE" OR “backache” OR “back pain” OR DE "SCIATICA" OR “sciatica” OR “Failed Back Surgery Syndrome” OR “DE "PIRIFORMIS syndrome" OR "PIRIFORMIS syndrome" OR DE "INTERVERTEBRAL disk displacement" OR "INTERVERTEBRAL disk displacement" OR DE "INTERVERTEBRAL disk hernias" OR “INTERVERTEBRAL disk hernias” OR “Spondylarthritis” OR “Spondylosis” OR "Coccydynia" OR “coccyx pain” OR "MYOFASCIAL pain syndromes" OR "MYOFASCIAL pain syndromes" OR “Sacroiliac Joint Dysfunction” OR “discogenic pain” OR “Intervertebral Displacement” OR “lumbago” OR "lower back pain" OR “Vertebrogenic Pain Syndrome” OR “back disorder” OR "lumbar spine pain" OR “sciatic neuralgia” OR “dorsalgia” OR “sciatic neuropathy” OR "dis* degradation" OR "degenerative intervertebral dis*" OR "dis* degeneration" OR "intervertebral dis* injury" OR "loin pain" OR "lumbosacral pain" OR "dis*ogenic pain" OR "dis* herniation" OR "ischialgia" OR "intervertebral dis* disease" OR "spinal fusion" OR "spondylolisthesis" OR "dis*itis" OR "spinal disease" OR "spinal osteochondrosis" OR "spine disease" OR "spinal osteophytosis" OR "spondylolysis" OR "spinal stenosis" OR "spondylitis" OR "dis*ectomy" OR "spondylarthritis" OR "lumbal pain" OR "lumbodynia" OR "lumbosacroiliac pain" OR "intervertebral dis* degeneration" OR "posture disorder" OR "spine abnormality" OR "kyphosis" OR "scoliosis” OR "sacrocoxalgia" OR "vertebral canal stenosis" OR DE "PELVIC pain" OR "pelvi* pain" OR (DE "BURSITIS") OR “bursitis” OR "hip pain" OR "thigh pain" OR DE "Knee Pain" OR "knee pain" OR "ankle pain" OR "foot pain" OR "toe pain" OR "heel pain" OR "foot pain" OR "joint pain" OR "muscle pain" OR "myalgia” Or DE "MYALGIA") OR "upper limb pain" OR "lower limb pain" OR "leg pain OR DE "ARTHRITIS" OR "ARTHRITIS" OR "arthralgia" OR “arthropathy” OR “ arthrochondritis” OR “arthrosynovitis” OR DE "TENDINOPATHY" OR "TENDINOPATHY" OR "tendonitis" OR "tenosynovitis" OR "bone pain" OR "ligament pain" OR "capsulitis" OR "synovitis" OR DE "MYOFASCIAL pain syndromes" OR “MYOFASCIAL pain syndromes" OR "myofascial pain" OR "impingement" OR DE "Patellofemoral Pain Syndrome" OR "Patellofemoral Pain" OR DE "Fasciitis" OR "fasciitis" OR "musculoskeletal pain" OR “Widespread pain” OR “spine fusion” OR “spine disease” OR “periarthritis”) | (DE "PHYSICAL therapy" OR DE "AQUATIC exercises" OR DE "MEDICAL rehabilitation" OR DE "NEUROPSYCHOLOGICAL rehabilitation" OR DE "MANIPULATION therapy" OR DE "COGNITIVE therapy" OR DE "RESISTANCE training" OR DE "PHYSICAL training & conditioning" OR DE "WEIGHT training" OR DE "PAIN management" OR DE "MOVEMENT therapy" OR DE "KINESIOLOGY" OR DE "HYDROTHERAPY" OR DE "FUNCTIONAL training" OR DE "PILATES method" OR DE "TAI chi" OR DE "BACK exercises" OR "PHYSICAL THERAPY" OR "AQUATIC EXERCISES" OR "NEUROPSYCHOLOGICAL REHABILITATION" OR "COGNITIVE THERAPY" OR "RESISTANCE TRAINING" OR "PHYSICAL TRAINING" OR "ENDURANCE TRAINING" OR "WEIGHT TRAINING" OR "PAIN MANAGEMENT" OR "MOVEMENT THERAPY" OR 'KINESIOLOGY" OR "MEDICAL REHABILITATION" OR "COGNITIVE BEHAVIO*RAL THERAPY" OR "CBT" OR "HYDROTHERAPY" OR "MUSCLE STRETCHING' OR "FUNCTIONAL TRAINING" OR 'BACK EXERCISES" OR "REHABILITATION" OR "BEHAVIO*R THERAPY" OR 'MUSCLE STRENGTHENING" OR 'THERAPEUTIC EXERCISE" OR "PHYSIOTHERAPY" OR "EDUCATION" OR "COGNITIVE PSYCHOTHERAPY" OR "COGNITION THERAPY" OR "MINDFULNESS" OR "ACCEPTANCE AND COMMITMENT THERAPY" OR "NEURODYNAMICS" OR "PILATES" OR "TAI CHI" OR "AQUATIC THERAPY" OR "MULTIDISCIPLINARY PAIN PROGRAM*" OR "BACK SCHOOL") | ("ALLODYNIA" OR "HYPERESTHESIA" OR "PAIN THRESHOLD" OR "ALLODYNIA" OR "QUANTITATIVE SENSORY TESTING" OR " PRESSURE PAIN THRESHOLD" OR "TEMPORAL SUMMATION" OR "CONDITIONED PAIN MODULATION" OR "CENTRAL SENSITI*ATION INVENTORY" "PUNCTATE SHARPNESS THRESHOLD" OR "MECHANICAL PAIN SENSITIVITY" OR "DETECTION THRESHOLD" OR "PAIN SENSITIVITY QUESTIONNAIRE" OR "PPT" OR "QST" OR "CENTRAL PAIN MODULATION" OR "NEURAL INHIBITION" OR "CENTRAL NERVOUS SYSTEM SENSITI*ATION" OR PAIN PERCEPTION" OR "SENSORY THRESHOLD" OR "OXYESTHESIA" OR "PAIN SENSITI*ATION" OR "CENTRAL SENSITI*ATION" OR "PERIPHERAL SENSITI*ATION" OR "ALGOMETRY" OR "HEAT PAIN" OR "COLD PAIN" OR "PAIN AMPLIFICATION" OR "PSYCHOPHYSICAL TESTING" OR "COLD HYPERALGESIA" OR "HEAT HYPERALGESIA" OR "VIBRATION DETECTION" OR "THERMAL HYPERALGESIA" OR "STATIC LIGHT TOUCH" OR "BLUNT PRESSURE" OR "PINPRICK" OR "NOCICEPTIVE FLEXION REFLEX" OR "SENSATION DETECTION THRESHOLD" OR "SPATIAL SUMMATION") |

**Search Strategy for Scopus Database**

| **Population** | **Intervention** | **Measurement tool** |
| --- | --- | --- |
| ( TITLE-ABS-KEY ( ( {Neck Pain}  OR  {cervical pain}  OR  {Whiplash Injuries}  OR  {whiplash}  OR  {Facial Pain}  OR  {face pain}  OR {jaw pain}  OR  {Shoulder Pain}  OR  {scapular pain}  OR  {arm pain}  OR  {Elbow Pain}  OR  {Tennis Elbow}  OR  {epicondyl*}  OR  {Golf Elbow}  OR  {golfers elbow}  OR  {wrist pain}  OR  {hand pain}  OR  {finger pain}  OR  {carpometacarpal joint}  OR  {metacarpal pain}  OR  {thora* pain}  OR  {thoracalgia}  OR  {rib pain}  OR  {chondritis}  OR  {Pelvic Pain}  OR  {Bursitis}  OR  {hip pain}  OR  {thigh pain}  OR  {knee pain}  OR  {ankle pain}  OR  {toe pain}  OR  {heel pain}  OR  {joint pain}  OR  {foot pain}  OR  {muscle pain}  OR  {myalgia}  OR  {upper limb pain}  OR  {lower limb pain}  OR  {leg pain}  OR  {arthritis}  OR  {arthralgia}  OR  {arthropathy}  OR  {arthrochondritis}  OR  {arthrosynovitis}  OR  {Tendinopathy}  OR  {tendonitis}  OR  {tenosynovitis}  OR  {bone pain}  OR  {ligament pain}  OR  {Bursitis}  OR  {capsulitis}  OR  {Synovitis}  OR  {Myofascial Pain Syndromes}  OR  {myofascial pain}  OR  {impingement}  OR  {Patellofemoral Pain Syndrome}  OR  {Fasciitis}  OR  {musculoskeletal pain}  OR  {Widespread pain}  OR  {spine fusion}  OR  {spine disease}  OR  {periarthritis}  OR  {Sacroiliac Joint Dysfunction}  OR  {low back pain}  OR  {back pain}  OR  {backpain}  OR  {sciatica}  OR  {failed back surgery syndrome}  OR  {piriformis syndrome}  OR  {Intervertebral Displacement}  OR  {Spondylosis}  OR  {Coccydynia}  OR  {Myofascial Pain Syndrome}  OR  {lumbago}  OR  {lower back pain}  OR  {back ache}  OR  {backache}  OR  {Vertebrogenic Pain Syndrome}  OR  {back disorder}  OR  {lumbar spine pain}  OR  {sciatic neuralgia}  OR  {dorsalgia}  OR  {sciatic neuropathy}  OR  {dis* degradation}  OR  {degenerative intervertebral dis*}  OR  {dis* degeneration}  OR  {intervertebral dis* injury}  OR  {loin pain}  OR  {lumbosacral pain}  OR  {dis*ogenic pain}  OR  {dis* herniation}  OR  {ischialgia}  OR  {intervertebral dis* disease}  OR  {lumbar pain}  OR  {spinal fusion}  OR  {spondylolisthesis}  OR  {dis*itis} OR  {spinal disease}  OR  {spinal osteochondrosis}  OR  {spine disease}  OR  {spinal ostephytosis}  OR  {spondylolysis}  OR  {spinal stenosis}  OR  {spondylitis}  OR  {dis*ectomy}  OR  {spondylarthritis}  OR  {lumbal pain}  OR  {lumbodynia}  OR  {lumbosacroiliac pain}  OR  {intervertebral dis* hernia}  OR  {intervertebral dis* degeneration}  OR  {posture disorder}  OR  {spine abnormality}  OR  {kyphosis}  OR  {scoliosis}  OR  {spondylodis*itis}  OR  {sacrocoxalgia}  OR  {vertebral canal stenosis} ) ) ) | ( TITLE-ABS-KEY ( ( {Physical Therapy}  OR  {Pain Management}  OR  {back exercise}  OR  {endurance training}  OR  {Cognitive Therapy}  OR  {Cognitive Psychotherapy}  OR  {Cognitive Behavio*ral therapy}  OR  {CBT}  OR  {Movement therapy}  OR  {Neuropsychological rehabilitation}  OR  {Behavio*r Therapy}  OR  {Resistance Training}  OR  {Muscle Strengthening}  OR  {Therapeutic Exercise}  OR  {Kinesiology}  OR  {Hydrotherapy}  OR  {Functional Training}  OR  {physiotherapy} OR  {medical rehabilitation}  OR  {education}  OR  {cognition therapy}  OR  {mindfulness}  OR  {acceptance and commitment therapy}  OR  {neurodynamics}  OR  {muscle stretching}  OR  {pilates}  OR  {tai chi}  OR  {aquatic therapy}  OR  {aquatic exercise}  OR  {physical training}  OR  {weight} OR  {training}  OR  {multidisciplinary pain program*}  OR  {back school}  OR  {Rehabilitation} ) ) ) | ( TITLE-ABS-KEY ( ( {allodynia}  OR  {hyperesthesia}  OR  {Pain Threshold}  OR  {quantitative sensory testing}  OR  {pressure pain threshold}  OR  {ppt}  OR  {qst}  OR  {temporal summation}  OR  {conditioned pain modulation}  OR  {central sensiti*ation inventory}  OR  {punctate sharpness threshold}  OR  {fibromyalgia impact questionnaire}  OR  {mechanical pain sensitivity}  OR  {detection threshold}  OR  {pain sensitivity questionnaire}  OR  {central pain modulation}  OR  {neural inhibition}  OR  {central nervous system sensiti*ation}  OR  {pain perception}  OR  {sensory threshold}  OR  {oxyesthesia}  OR  {pain sensiti*ation}  OR  {Central Sensiti*ation}  OR  {peripheral sensiti*ation}  OR  {algometry}  OR  {heat pain}  OR  {cold pain}  OR  {pain amplification}  OR  {psychophysical testing}  OR  {cold hyperalgesia}  OR  {heat hyperalgesia}  OR  {vibration detection}  OR  {thermal hyperalgesia}  OR  {static light touch}  OR  {blunt pressure}  OR  {pinprick}  OR  {nociceptive flexion reflex}  OR  {sensation detection threshold}  OR  {spatial summation} ) ) ) |
